# Study of Anion Exchange Membrane Properties Incorporating *N*-spirocyclic Quaternary Ammonium Cations and Aqueous Organic Redox Flow Battery Performance

**DOI:** 10.3390/membranes11050367

**Published:** 2021-05-18

**Authors:** Misgina Tilahun Tsehaye, Xian Yang, Tobias Janoschka, Martin D. Hager, Ulrich S. Schubert, Fannie Alloin, Cristina Iojoiu

**Affiliations:** 1 Univ. Grenoble Alpes, Univ. Savoie Mont Blanc, CNRS, Grenoble INP, LEPMI, 38 000 Grenoble, France; misgina-tilahun.tsehaye@grenoble-inp.fr; 2 Laboratory of Organic and Macromolecular Chemistry (IOMC), Friedrich Schiller University Jena, Humboldtstrasse 10, 07743 Jena, Germany; xian.yang@jenabatteries.de (X.Y.); martin.hager@uni-jena.de (M.D.H.); ulrich.schubert@uni-jena.de (U.S.S.); 3 Center for Energy and Environmental Chemistry Jena (CEEC Jena), Friedrich Schiller University Jena, Philosophenweg 7a, 07743 Jena, Germany; 4 JenaBatteries GmbH, Otto-Schott-Strasse 15, 07745 Jena, Germany; 5 Réseau sur le Stockage Electrochimique de l’Energie (RS2E), CNRS, FR3459, CEDEX, 80 039 Amiens, France

**Keywords:** anion exchange membrane, poly (diallylpiperidinium chloride), TMA-TEMPO/MV, aqueous organic redox flow battery, cyclability

## Abstract

Flexible cross-linked anion exchange membranes (AEMs) based on poly (*p*-phenylene oxide) grafted with *N*-spirocyclic quaternary ammonium cations were synthesized via UV-induced free-radical polymerization by using diallylpiperidinium chloride as an ionic monomer. Five membranes with ion exchange capacity (IEC) varying between 1.5 to 2.8 mmol Cl^−^·g^−1^ polymer were obtained and the correlation between IEC, water uptake, state of water in the membrane and ionic conductivity was studied. In the second part of this study, the influence of properties of four of these membranes on cell cycling stability and performance was investigated in an aqueous organic redox flow battery (AORFB) employing dimethyl viologen (MV) and *N*,*N*,*N*-2,2,6,6-heptamethylpiperidinyl oxy-4-ammonium chloride (TMA-TEMPO). The influence of membrane properties on cell cycling stability and performance was studied. At low-current density (20 mA·cm^−2^), the best capacity retention was obtained with lower IEC membranes for which the water uptake, freezable water and TMA-TEMPO and MV crossover are low. However, at a high current density (80 mA·cm^−2^), membrane resistance plays an important role and a membrane with moderate IEC, more precisely, moderate ion conductivity and water uptake was found to maintain the best overall cell performance. The results in this work contribute to the basic understanding of the relationship between membrane properties and cell performance, providing insights guiding the development of advanced membranes to improve the efficiency and power capability for AORFB systems.

## 1. Introduction

Redox flow batteries (RFBs) represent promising large-scale energy storage devices for many reasons, such as, being a source of independently scalable power and energy, allowing for modular production, are operational at ambient temperatures and are environmentally benign [1,2,3,4]. In recent years, organic redox flow batteries (ORFBs) have grown to become very promising candidates to fulfill the requirements of ‘‘green’’, safe and sustainable energy storage [5]. Among them, aqueous ORFBs (AORFBs), which use water as solvent, are of high interest for both industry and academia [6,7,8,9,10,11]. Particularly, the neutral pH value AORFB employing water-soluble dimethyl viologen (MV, *N*,*N*’-dimethyl-4,4′-bipyridinium dichloride) (anolyte) and TMA-TEMPO (*N*,*N*,*N*-2,2,6,6-heptamethylpiperidinyl oxy-4-ammonium chloride) (catholyte) molecules has been demonstrated as a stable and high performing flow battery system [12]. A relatively high capacity of 54 Ah·L^−1^ giving a total energy density of 38 Wh·L^−1^ at a cell voltage of 1.4 V has been reported using TMA-TEMPO (2.3 M)/MV (2.4 M) as the active materials [12]. The relatively high energy density of this system is due to the high solubility of both active materials. Furthermore, the solubility of the organic active materials and, hence, energy density can be further improved by modifying the functional group or tuning the composition of the supporting electrolytes [13,14].

Over the years, most research efforts have been focused on redox-active organic compounds [15,16]. However, the membrane separating catholyte and anolyte has been identified as one of the major obstacles in the deployment of various RFBs [17]. Battery performance is strongly defined by the membrane, since the key role of the membrane is to avoid the mixing of redox-active species and to conduct charge-carrying ions (e.g., Cl^−^ ion for TMA-TEMPO/MV system) for the electrochemical reaction. In other words, for a high power density battery, a membrane with high ionic conductivity is required, while for a high coulombic efficiency and capacity retention, the membrane should avoid the cross-contamination of active species [18,19]. Additionally, a well performing membrane should possess certain properties: (i) low ohmic resistance, (ii) good chemical stability, (iii) good mechanical stability and (iv) acceptable cost [5,20,21,22]. Moreover, the transport phenomena through a membrane used in RFBs are more complex and challenging as compared to those involved in other broadly studied systems such as fuel cells, as more ionic species are involved in transport activities driven by diffusion, osmosis and migration [16]. Therefore, to optimize the cell performance and to develop adapted membranes, there is a need to understand the correlation between polymer structure/membrane properties/transport phenomena and its influence on the overall cell performance. After all, it is the polymer structure (backbone, ionic moieties, ion exchange capacity (IEC), etc.) and microstructure of the membrane that endow the membrane with the different properties.

To date, the state-of-the-art ORFB systems mostly use commercial membranes developed for other applications [12,23,24,25,26] that are not optimized for this type of flow battery. Moreover, different ORFB systems require different types of membranes. For the TMA-TEMPO/MV system, an anion exchange membrane (AEM) is suitable to conduct the charge using carrier–chloride ions. The influence of membrane properties (based on commercial membranes) on ORFB cell performance has been reported in the literature [27,28]. In 2017, Bo Hu et al. [27] performed a systematic study of the effects of commercial ion exchange membranes (Selemion^®^ AMV, DSV, ASV) and supporting electrolytes (NaCl, KCl) on the cell resistance and cell performance of a neutral AORFB (FcNCl (ferrocenylmethyl) trimethyl-ammonium chloride)/MV). Results showed that the thinnest membrane DSV with the lowest area resistance displayed the best current-dependent performance, energy efficiency, capacity utilization and power density. This work emphasized the internal battery resistance as the primary factor to reach high battery performance. However, the crossover issue was neglected and the potential impact of the membrane structure was not studied. In 2019, Small et al. [28] compared the performances of five different commercial AEMs (AFX, AHA, AMV, ASV, DSV) in a TEMPO/MV-based ORFB. Taking into consideration the cumulative contributions of pressure-driven flow, osmosis of solvent and redox-active species, migration and the electroosmotic drag, capacity loss with each membrane was traced back to their corresponding water content and IEC. However, since different commercial membranes (different polymer, cation, design and preparation procedures) were used, it is difficult to solely attribute the variation to the IEC of the membranes.

In this work, poly(phenylene oxide) (PPO) was chosen as polymer backbone because of its good mechanical strength, good chemical stability and commercial availability in large quantities [29]. Moreover, it offers versatile synthetic routes and can be modified by easily controlling the degree of functionalization to meet various application requirements [30]. Recently, *N*-spirocyclic quaternary ammonium (QA) cations have been reported to be more chemically stable against elimination and ring-opening substitution reactions than other ammonium species due to their geometric constraints, such as unfavorable bond angles and lengths in the reaction transition states [31]. However, incorporating those *N*-spirocyclic QAs into the polymer backbone is a considerable challenge in the synthesis of AEMs bearing such cations [32,33].

Herein, we first provide a simple and convenient synthetic method to incorporate the *N*-spirocyclic QA to the membrane structure via a rapid UV-irradiation approach. The PPO polymer backbone was first functionalized with methylbenzyldiallylammonium groups (PPO-Q) via a two-step process and then cross-linked in the presence of *N*,*N*-diallylpiperidinium chloride (DAPCl) via UV-induced free-radical polymerization. *N*-spirocyclic QA cation was chosen to minimize the possible chemical degradation of the membranes as radical active species are involved in the discharge/charge process of the battery. In order to go deeper into the understanding of the structure/properties relationships, five membranes with different IECs were synthetized by varying the feed ratio of the DAPCl and PPO-Q. Subsequently, the membranes were characterized in terms of IEC, thickness, water uptake and chloride ion conductivity before they were used in battery test cells. Moreover, for the assessment of the reproducibility of the improved synthetic method, three samples of each membrane type were tested in the TMA-TEMPO/MV-based ORFB system and their charge/discharge cycling performance was recorded. Taking into account both cell resistance and cross-contamination, the effect of membrane properties (IEC, water uptake, chloride conductivity and thickness) on the corresponding cell performance (capacity utilization, coulombic efficiency, energy efficiency and power density) were discussed.

## 2. Materials and Methods

All chemicals, if not specified otherwise, were used as received without further purification. Poly (2,6-dimethyl-1,4-phenyleneoxide) (PPO) (*M*_n_ = 20,000, *Đ* = 2.5) was purchased from Polysciences Inc. and dried under vacuum at 60 °C overnight before use. Methanol (99.9%), ethanol (99.9%) and chloroform (99.8%) were purchased from Fisher Scientific. *N*-Bromosuccinimide (NBS) (99%), 2,2′-azobis(2-methylpropionitrile) (AIBN) (98%), tetrahydrofuran (THF, ACS, >99%), diethyl ether (>99%), chloroform-d (CDCl_3_-d, 99.9% D), Irgacure^®^ 2959, D_2_O (99.9% D) and 1,2-dichloroethane (99.8%) were purchased from Sigma-Aldrich (Merck KGaA, Darmstadt, Germany). Dimethyl sulfoxide-d_6_ (DMSO-d_6_, 99.9%), *N*-methyl-2-pyrrolidone (NMP, reagent grade) were supplied from Acros Organics. Chlorobenzene (ACS reagent, ≥99.5%), diallymethylamine (97%) and piperidine (≥99%) were bought from ABCR GmbH. Allyl bromide (98%) and allyl chloride (98%) were bought from Alfa Aesar. The electrolytes MV and TMA-TEMPO were provided by JenaBatteries GmbH (Jena, Germany). FAA-3-50^®^ was purchased from Fumatech BWT GmbH (Bietigheim-Bissingen, Germany).

### 2.1. Polymer Synthesis

Brominated PPO (PPO-Br): In order to introduce the *N*-spirocyclic QA cation, commercial PPO (*M*_n_ = 20,000, *M*_w_/*M*_n_ = 2.5) was first functionalized by free-radical bromination, using NBS as a brominating agent and AIBN as an initiator [34]. In a two-neck round bottom flask equipped with a condenser, PPO (6 g, 50 mmol repeating units) was dissolved in chlorobenzene (60 mL) under stirring. Then NBS (2.07 g, 11.6 mmol) and AIBN (0.115 g, 0.7 mmol) were added. The reaction mixture was heated at 136 °C under reflux for 3 h, in order to ensure benzylic bromination [35]. Afterwards, the mixture was poured into an excess of methanol (600 mL) to form a light-brown precipitate of brominated PPO (PPO-Br). The PPO-Br polymer was filtered, dried overnight in a vacuum oven at 60 °C and re-dissolved in chloroform (60 mL), followed by precipitation in a 10-fold excess of ethanol. The precipitate was dried in a vacuum oven at 60 °C for 24 h before next use. The extent of bromination was calculated based on the ^1^H NMR spectrum by comparing the integrals of the brominated methylene at 4.3 ppm and aromatic methyl group at 2.1 ppm. ^1^H NMR (400 MHz, CDCl_3_) *δ* 6.61 (m, 2H), 6.43 (m, 2H), 4.27 (s, 2H), 2.06 (s, 9H).

**Scheme 1:**
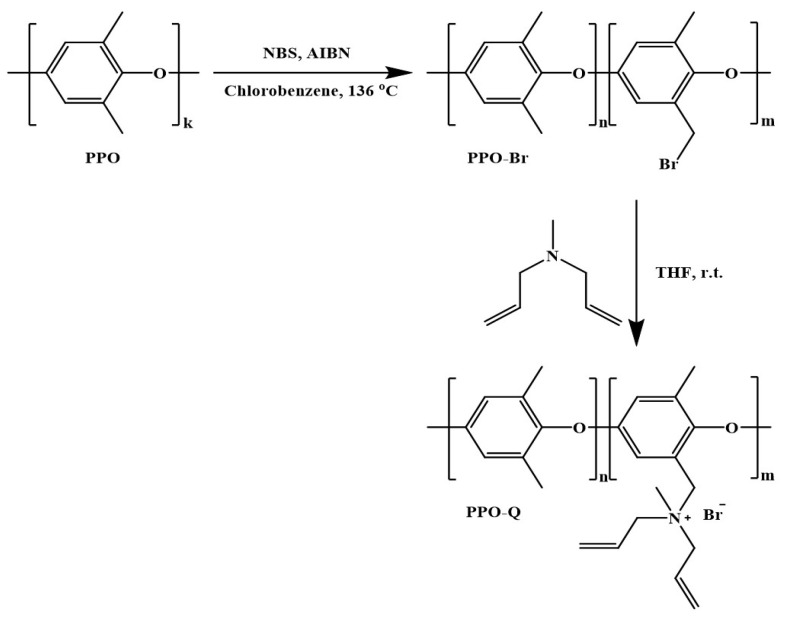
Schematic representation of the preparation of PPO-Q polymer (m = 0.15 and n = 0.85).

Quaternized PPO (PPO-Q): The quaternized PPO (PPO-Q) polymer was prepared by reacting PPO-Br with diallylmethyllamine [36]. Diallylmethylamine (molar ratio diallylmethylamine to Br in PPO-Br = 2.5) was added to 10 wt.% PPO-Br and dissolved in THF solution under argon at room temperature. A long reaction time (48 h) was used to assure the complete reaction. The quaternized product was precipitated in diethyl ether and dried in a vacuum oven at 35 °C for 48 h. The successful substitution of the Br atoms by diallylmethylamine and quaternization of the product was confirmed by ^1^H NMR. ^1^H NMR (400 MHz, DMSO) *δ* 6.76 (d, *J* = 201.6 Hz, 1H), 6.07 (s, 2H), 5.49 (d, *J* = 58.5 Hz, 4H), 4.29 (s, 2H), 3.91 (s, 4H), 2.88 (s, 3H), 2.02 (s, 9H). The reaction protocol is summarized in Scheme 1.

### 2.2. N,N-Diallylpiperidinium Chloride (DAPCl)

*N*-Allylpiperidine [32]: In a 250 mL reaction flask, sodium hydroxide (14.5 g, 0.36 mol) was dissolved in deionized water (20 mL) at 0 °C and piperidine (30 mL, 0.30 mol) was added to the solution. Finally, allyl bromide (26.3 mL, 0.30 mol) was added dropwise (ca. 30 min). The temperature was raised to room temperature and the solution stirred for 6 h. The oil layer was collected using a separatory funnel and the remaining aqueous layer was extracted once with chloroform. The chloroform was removed via distillation. The product was washed with deionized water until pH = 7 was reached. Finally, the product was dried over MgSO_4_ and filtered. The remaining colorless liquid (32.3 g, 86% yield) was collected. ^1^H NMR (400 MHz, CDCl_3_) *δ* 5.69–5.53 (m, 1H), 4.95–4.78 (m, 2H), 2.72–2.65 (m, 2H), 2.10 (s, 4H), 1.32 (dt, *J* = 11.3, 5.6 Hz, 4H), 1.17 (dd, *J* = 11.2, 5.8 Hz, 2H).

**Scheme 2:**
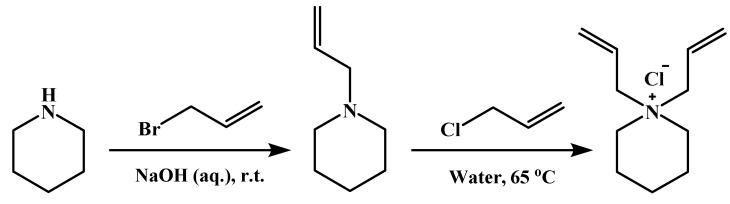
Schematic representation of the synthesis of *N*-allylpiperidine and *N*,*N*-diallylpiperidinium chloride.

*N*,*N*-Diallylpiperidinium chloride (DAPCl): *N*-Allylpiperidine (32.3 g, 0.258 mol), allyl chloride (26.3 mL, 0.323 mol) and water (50 mL) were added into a reaction flask equipped with a magnetic stirrer bar (Scheme 2). The reaction flask was sealed with a rubber septum secured by Cu wire. The mixture was stirred at 65 °C for 48 h. The oil layer was collected using a separatory funnel and the remaining aqueous layer was extracted once with diethyl ether. After solvent evaporation, the viscous oil was precipitated in acetone. The solid product was washed several times using diethyl ether and precipitated in acetone to collect a white solid product by filtration (32.4 g, 62% yield). ^1^H NMR (400 MHz, D_2_O) *δ* 6.14–5.97 (m, 2H), 5.73 (m, 4H), 3.95 (d, *7*.2 Hz, 4H), 3.37 (m, 4H), 1.96 (s, 4H), 1.77–1.64 (m, 2H).

### 2.3. Membranes Preparation

Five membranes with different expected IECs ranging from 1.96 to 3.25 mmol Cl^−^·g^−1^ polymer (Table 1) were prepared and characterized. To manipulate the IEC, the DAPCl to PPO-Q ratio was tuned. In a typical example, a DAPCl to PPO-Q ratio of two (referred as ‘M1.7’) was used to prepare an AEM with a theoretical IEC of 2.23: 0.075 g PPO-Q (0.076 mmol diallylmethylammonium), 0.0306 g DAPCl (0.152 mmol) and 0.01023 g (20% excess with respect to the diallylmethylammonium in the polymer) initiator (Irgacure^®^ 2959) were dissolved in 1.2 mL of 1,2-dichloroethane (13 wt/v.%). After addition of 0.41 mL NMP (28.5 wt/v. %), the mixture was stirred for ten minutes. The thicknesses of the obtained membranes were between 39 and 60 microns.

**Figure 1:**
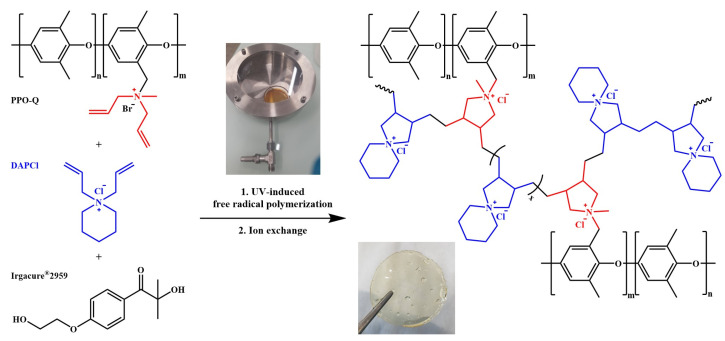
Schematic representation of the membrane preparation procedure.

The solution was filtered and poured into a petri dish with 5 cm diameter. The glass petri dish was kept away from light to avoid photodecomposition of the initiator. After almost complete evaporation of the 1,2-dichloroethane under a fume hood at room temperature after ca. 30 min, the petri dish containing the viscous solution was put in a vacuum chamber (Figure 1) and degassed in order to remove oxygen. The film was then cross-linked using UV radiation (P300MT power supply, Fusion UV Systems) with a UV light exposure for 3 min. The obtained membrane was kept in a vacuum oven to evaporate the residual solvent at 60 °C for 24 h.

### 2.4. Ex Situ Membrane Characterization

#### 2.4.1. Structural Characterization

The structure of the polymers and QA cation was determined by ^1^H NMR spectroscopy. A Bruker AV 400 NMR spectrometer was used to record the ^1^H NMR spectra. DMSO-d_6_ (*δ* = 2.50 ppm) or CDCl_3_ (*δ* = 7.26 ppm) or D_2_O (*δ* = 4.79 ppm) were used as solvents.

#### 2.4.2. Membrane Chloride Conductivity

Sample preparation: The membranes were immersed in aqueous NaCl solution (1 M) at room temperature for 24 h to exchange bromide ions for chloride ions. Afterwards, they were immersed, and slowly stirred, in excess deionized water overnight to remove the excess of salts. Finally, they were rinsed with deionized water.

Measurement: The conductivity of the membranes was measured at room temperature via a through-plane impedance technique using a homemade measuring cell (Figure S1) and a Hewlett Packard Model Frequency response analyzer (HP Model 4192A LF Impedance Analyzer, Yokogawa Hewlett Packard, Japan) by two-point probe impedance method in the AC frequency range 13 MHz to 5 Hz. The membrane resistance is obtained as the high-frequency intersected by the real impedance axis in the Nyquist plot. The conductivity was calculated using Equation (1):

(1) $$\sigma =\frac{L}{R\times A}$$

where σ is the ionic conductivity (S·cm^−1^), *L* (cm) is the distance between the electrodes (wet membrane thickness), *R* (Ω) is the resistance of the membrane obtained at high frequency and *A* (cm^2^) is the membrane area (0.0314 cm^2^). The conductivity for each membrane was reported as the average of at least three measurements.

#### 2.4.3. Ionic Exchange Capacity and Water Uptake

Sample preparation: The membranes were converted to Cl^−^ form by soaking in aqueous NaCl (1 M) solution for 48 h. Afterwards, excess salt was removed by washing the membranes approximately seven times with deionized water.

Measurement: The *IEC*, meaning the number of charged functional groups per gram of polymer, was measured using the Mohr titration method [37,38]. Membrane samples (~0.05 g) were dried in a vacuum oven at 50 °C for 24 h. The dry weight of the samples was recorded before immersion in 0.5 M aq. Na_2_SO_4_ (25 mL) for at least 48 h under stirring, thus replacing the Cl^−^ with ${\mathrm{SO}}_{4}^{2-}$. Three samples of the resulting solution (containing released Cl^−^ ions) were titrated with 0.01 M aq. AgNO_3_. K_2_CrO_4_ was used as an indicator. The *IEC* of the membranes was calculated using the following Equation (2):

(2) $$IEC\;\left(\mathrm{mmol}\cdot g^{-1}\right)=\frac{Volume\;of\;{\mathrm{AgNO}}_{3}\;\left(\mathrm{mL}\right)\times Concentratio\;of\;{\mathrm{AgNO}}_{3}\left(\mathrm{mmol}\cdot {\mathrm{mL}}^{-1}\right)}{Weight\;of\;dry\;membrane\;\left(g\right)}$$

Water uptake (*WU*) of the membrane after immersion in water at room temperature for 24 h was calculated using the weight of the dry and wet membrane samples. The *WU* of the membrane was calculated using Equation (3):

(3) $$WU\left(\%\right)=\frac{W_{wet}-W_{dry}}{W_{dry}}\times 100$$

The hydration number (or membrane water concentration) (λ), defined as the number of water molecules per (functional) ion exchange group, is estimated as follows (Equation (4)):

(4) $$\lambda =\left(\frac{W_{wet}-W_{dry}}{18.01}\right)\left(\frac{1000}{IEC\times W_{dry\;}}\right)=\frac{WU\;\left(\%\right)\times 10}{IEC\times 18.01}\;$$

The water uptake, *IEC* and hydration number for each membrane were reported as the average of at least three measurements.

#### 2.4.4. Differential Scanning Calorimetry Experiments

Differential scanning calorimetry (DSC) measurements were carried out using a Mettler-Toledo DSC1 instrument in order to determine the freezable (*N_free_*) and non-freezable (*N_non_*) water molecules in the membranes. Membrane samples were immersed in deionized water for at least a week to fully hydrate them. The samples (about 10 mg) were surface-dried with tissue paper and quickly sealed in aluminum pans. They were analyzed in thermal cycles of cooling from 25 to −50 °C, followed by stabilization at −50 °C for 10 min and successively heating up to room temperature under N_2_ gas (50 mL·min^−1^) at a scan rate of 5 K·min^−1^. The amount of freezable (ratio of the mass of freezable water to the total mass of water in the membrane) and non-freezable water molecules was calculated using Equations (5) and (6) [39,40]:

(5) $$N_{free}=\frac{M_{free}}{M_{tot}}\times \lambda =\frac{\Delta H_{free}/\Delta H_{ice}}{M_{tot}}\times \lambda$$

where *M_free_* and *M_tot_* are the masses of freezable and total water molecule absorbed in the membrane. Δ*H*_f_ is the enthalpy obtained by the integration of the DSC freezing peak and Δ*H*_ice_ is the enthalpy of fusion for water (334 J·g^−1^). The non-freezable bound water (*N_non_*) was estimated by subtracting the freezable water content from the total hydration number.

(6) $$N_{non}=\lambda -N_{free}\;$$

#### 2.4.5. Thermal Analysis

The thermal stability of the monomer, polymer and membranes were measured by using a thermogravimetric analysis instruments TGA Q500 (Mettler-Toledo AG). Prior to analysis, the samples were dried at room temperature under vacuum for at least 24 h. The samples were preheated at 100 °C for 30 min in the TGA to remove traces of water. The measurement was performed under a N_2_ atmosphere from 25 to 600 °C at a heating rate of 10 K·min^−1^.

#### 2.4.6. Microscopy

The morphologies of the membranes before and after cell test were checked using optical microscopy (Carl Zeiss Microscopy GmbH, Jena, Germany) in order to detect potential morphological changes. The membrane samples were wetted with water and then placed under the camera lens. The studied area of the sample was roughly divided into five regions, four corners and the center. All regions were thoroughly examined.

### 2.5. Flow Battery Test

#### 2.5.1. Charge/Discharge Tests

**Figure 2:**
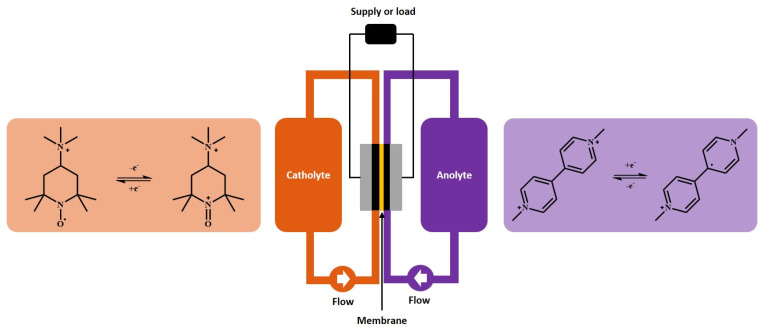
Schematic representation of the working principle of a redox flow battery employing solutions of TMA-TEMPO and MV. Chloride is used as a counter ion.

All cell tests carried out in this study were based on a lab scale single cell modified from the setup reported previously (JenaBatteries GmbH, Jena, Germany, active area: 5 cm^2^) [41] using a VMP3 potentiostat/galvanostat (BioLogic, Seyssinet-Pariset, France). The TMA-TEMPO/MV-based ORFB system is schematically shown in Figure 2. The redox-active species TMA-TEMPO and MV are dissolved in water and the electrolyte solutions are pumped through carbon felt electrodes. The membrane is sandwiched between the two electrodes to selectively bypass the conducting chloride ions and retain TMA-TEMPO/MV. For each membrane type, three samples were tested. Due to the complexity of the battery system, a commercial membrane FAA-3-50^®^ was selected as reference material to compare the cell performance with the prepared membranes.

A representative protocol of the charging/discharging test is described as follows: membranes were cut to squares of 3 cm × 3 cm and immersed in 0.5 M NaCl aqueous solution for 24 h before use. TMA-TEMPO (aq.) (10 mL, 1.12 mol·L^−1^) as catholyte and MV (aq.) (10 mL, 1.49 mol·L^−1^) as anolyte were used. Peristaltic pump (Heidolph Pumpdrive 5201, MASTERFLEX pump) was used for electrolyte circulation with a flow rate of 16 mL·min^−1^ in an air-conditioned room of 22 °C. The first charge/discharge cycle was run at a current density of 20 mA·cm^−2^. The cutoff voltages were 1.5 V and 0.9 V. Afterwards, it was charged/discharged at 80 mA·cm^−2^ for consecutive 100 cycles. The final two cycles were performed at 20 mA·cm^−2^.

Resistance: An empty cell was manufactured with electrolytes without the membrane and the resistance was recorded as *R_empty_* by electrochemical impedance spectroscopy (EIS) using the high-frequency real axis intercept in the Nyquist plot. The full cell resistances before and after the cycling tests were recorded by EIS and denoted as R_cell_. The membrane resistance per square centimeter was calculated by *R_mem_* (Ω·cm^2^) = (*R_cell_* − *R_empty_*) × active area. 

Efficiency: The coulombic efficiency (CE), voltage efficiency (VE) and energy efficiency (EE) were calculated with the following equations:

(7) $$\mathrm{CE}=\frac{t_{d}}{t_{c}}\times 100\%$$

(8) $$\mathrm{VE}=\frac{U_{d}}{U_{c}}\times 100\%$$

(9) EE= CE×VE

where t_d_ is the discharging time, t_c_ is the charging time, U_d_ is the average discharging voltage and U_c_ represents the average charging voltage. The same current was used for charging and discharging.

#### 2.5.2. Cyclic Voltammetry

After each charge/discharge experiment, the electrolyte solutions were characterized by cyclic voltammetry (CV) (VMP3 potentiostat/galvanostat Bio-Logic, France). The measurements were performed in 0.5 M NaCl (aq.) at room temperature in a three-electrode setup, where glassy carbon serves as working electrode, platinum wire as counter electrode, AgCl/Ag as reference electrode. The background CV was scanned first and then 100 µL of the investigated electrolyte solution was added into 4.6 mL NaCl (aq.) (0.5 M) under inert gas atmosphere. The potential was scanned six times within the range of 0.75 to 1.05 V at a scan rate of 200 mV·s^−1^. Background was subtracted and the fourth scan was chosen to prepare comparative graphs.

#### 2.5.3. Polarization Curves

Polarization curves were acquired directly after the cell cycling test without changing electrolytes. The following program was employed: The cell was fully charged at a constant current density of 50 mA·cm^−2^ until it reached the upper limiting voltage of 1.5 V and then discharged at 10 mA·cm^−2^ for 30 s. It was again charged at the same 50 mA·cm^−2^ to 1.5 V, then discharged for 30 s at 20 mA·cm^−2^. Every second cycle, the discharge current density was increased by 10 mA·cm^−2^ until it reached 600 mA·cm^−2^ or the lower limiting voltage of 0.2 V. The last point of each discharge curve was taken as the discharge cell voltage at the corresponding current density. The power density was calculated by multiplying the cell voltage by the current density.

## 3. Results and Discussion

### 3.1. Synthesis of the Membrane

Commercial PPO was brominated in the benzylic position. The bromination degree was calculated by analysis of ^1^H NMR spectrum from the ratio between the integrals of characteristic peak of protons of –CH_2_Br at 4.3 ppm and the remaining unbrominated CH_3_ groups (2.1 ppm) (Figure S2a). Moreover, the absence of a signal at 6.1 ppm indicated that no phenyl-brominated polymers were formed as side products. The bromination degree was calculated to be 15%, which corresponds to a bromination yield of 65%. In a second reaction step, the PPO-Br was reacted with diallylmethylamine to form quaternized PPO-diallylmethylamine bromide (PPO-Q). New signals corresponding to the protons of the diallylmethylammonium moiety appeared in the ^1^H NMR spectrum (Figure S2b). The ratio between the integrals of the backbone and the substituent signals confirm the complete transformation of CH_2_Br to quaternized ammonium group.

DAPCl was prepared via a two-step process by a nucleophilic substitution reaction as outlined in Scheme 2 [32]. The structure and purity of the monomer were confirmed by ^1^H NMR spectroscopy as well (Figure S3). Different amounts of DAPCl were copolymerized with the PPO-Q (Table 1) in order to vary the theoretical *IEC* between 1.96 and 3.3 mmol of Cl^−^·g^−1^ of polymer and to study its impact on membrane properties and cell performance. The blend of PPO-Q polymer, DAPCl monomer and photoinitiator was cast from a solution of 1,2-dichloroethane and NMP (Section 2.4). The photo-polymerization initiator Irgacure^®^ 2959, a highly efficient non-yellowing radical Type I photoinitiator for UV curing systems [42,43], was used to initiate the reaction. Irgacure^®^ 2959 was chosen as radical photoinitiator because of its solubility in 1,2-dichloroethane, good overlap of absorbance spectrum with the emission spectrum of the light source (visible light absorbance) and commercial availability. To assure a good yield of the crosslinking reaction, a good mobility of double bonds is required. Therefore, NMP was employed as a co-solvent (“plasticizer”) during the irradiation as 1,2-dichloroethane is a highly volatile solvent.

The dried membranes were washed with water to remove the unreacted monomers, non-grafted oligomers, remaining initiator and NMP. Amounts corresponding to 10 to 20 wt.% of the total membrane dry weight were removed. The ^1^H NMR spectra of the residues revealed the presence of a mixture of DAPCl and poly(DAPCl) in a molar ratio close to 50:50.

The experimental *IEC*s were determined by titration and are summarized in Table 2. From these values, and taking into account that only the DAPCl and poly(DAPCl) were removed from the cast membrane, the actual ratios between the polymer side chain and DAPCl monomer, which were converted by the crosslinking reaction, were calculated (Table 1). The DAPCl conversion varied between 46 and 66% and increased with the increase of *IEC*. This increase can be explained by the (i) lower viscosity and (ii) higher double bond concentration in the polymer/monomer blend before irradiation. Flexible and transparent membranes with thickness between 39 and 60 µm were obtained. Similarly, Strasser et al. [32] and Jannasch et al. [36] reported the preparation of water-insoluble, chemically stable and mechanically robust AEMs using poly(DAPCl) cation prepared via UV-initiated radical and reactive casting cyclo-polymerization, respectively.

### 3.2. Ex situ Membrane Characterization

#### 3.2.1. Thermal Stability

The thermal stability of the prepared polymers, cation and membrane (M2.8) was evaluated with thermogravimetric analysis (TGA) (Figure S4). The membrane reveals a high thermal stability, the weight loss associated with the degradation of M2.8 membrane was registered to be above 250 °C corresponding to the degradation of benzylic quaternary amine side chains. The second thermal degradation step of the membrane occurred at 320 °C, in agreement with the poly(DAPCl) degradation temperature [36]. The last step at about 440 °C corresponds to the degradation of the PPO polymer backbone [44] and additional degradation of the poly(DAPCl) side chain. Similar two-step degradation of the poly(DAPCl) cation was reported elsewhere [36]. Therefore, the membranes have shown good thermal stability, which is better than what is required in the battery, typically operated at room temperature.

#### 3.2.2. Water Uptake

Figure 2 shows the water uptake of the membranes (Cl^−^ form) at room temperature. At lower *IEC* values, the water uptake slightly increased between M1.5 and M1.7 membranes, an increase of only 5% is observed. Whereas, at higher *IEC*, a much sharper increase (more than 50%) in water uptake between M2.1 and M2.8 membranes was recorded. This sudden increase is due to (i) the increase of proportion of the hydrophilic ionic side chains in the membrane, i.e., the higher the *IEC*, the longer the lengths of poly(DAPCl) and (ii) the decrease of the crosslinking stiffness [45].

The hydration number values (λ, the number of absorbed water molecules per QA cation) of the membranes followed a similar trend. At relatively low *IEC*, λ is roughly the same in M1.5 and M1.7 membranes, averaging 11.6 and 11.8, respectively. As the *IEC* increases, the corresponding λ increased significantly (M2.1, ~14; M2.8, 21.5 water molecules). A similar hydration number, increasing with an increase of *IEC*, has been reported in the literature [46,47]. Based on our results, it can be assumed that for M1.5 and M1.7 membranes, the *IEC* drives water uptake by the interaction of water with the anions and cations, whereas in the other three membranes with higher *IEC*s, the osmotic pressure becomes too high, and water clusters are formed. It should be noted that cation hydration [48] corresponds to the amount of water molecules directly interacting with the cation, whereas hydration shell indicates the number of water molecules surrounding the ion, but not necessarily directly interacting with it [49,50]. For instance, the cation hydration of a chloride ion was reported to be six in water solution [51,52], whereas the cation hydration of poly(DAPCl) has not yet been reported. However, the λ of a PPO-based AEM which has the same structure as ours, in OH^−^ form (2.34 mequiv·g^−1^ *IEC* and 101% water uptake at 20 °C), was reported to be 25 [36].

To gain further information about the state of water, i.e., in interaction with ions or free water, we performed DSC measurements. Generally speaking, the water absorbed in the membrane can be classified into freezable and non-freezable water states [53]. The freezable or free state water has the physical characteristics of bulk water, including freezing temperature close to 0 °C. On the other hand, the non-freezable water interacts strongly with the ionic functionality or polymer polar groups of the membrane. DSC measurements were done to investigate the water states in four of the prepared membranes. As shown in Table 3, there seems to be non-freezable water of about 11.4 to 14.5 molecules per ionic functions of the prepared membranes. There were no freezable water molecules in the case of M1.5 membranes. For the other membranes, the freezable amount of water was found to increase in line with their water uptakes. However, the freezable water molecules of M1.7 and 2.1 membranes do not exceed 1, whereas within the M2.8 membrane the freezable water molecules were found to be 7 ± 0.8 per ionic function and melt at about 0 °C, indicating the bulk water behavior of part of the absorbed water molecules. For the other membranes, the melting occurs at lower temperatures, which can be associated with confinement or interaction effects in accordance with the weak amount of water molecules involved in the melting process. In conclusion, we expect that the membranes with higher amount of *N_free_* to result in a higher degree of cross-contamination of TMA-TEMPO and MV compared to the membranes with low or no *N_free_* as the *N_non_* is mainly associated with the ionic functions, and less available for active species mobility.

#### 3.2.3. Conductivity

**Figure 3:**
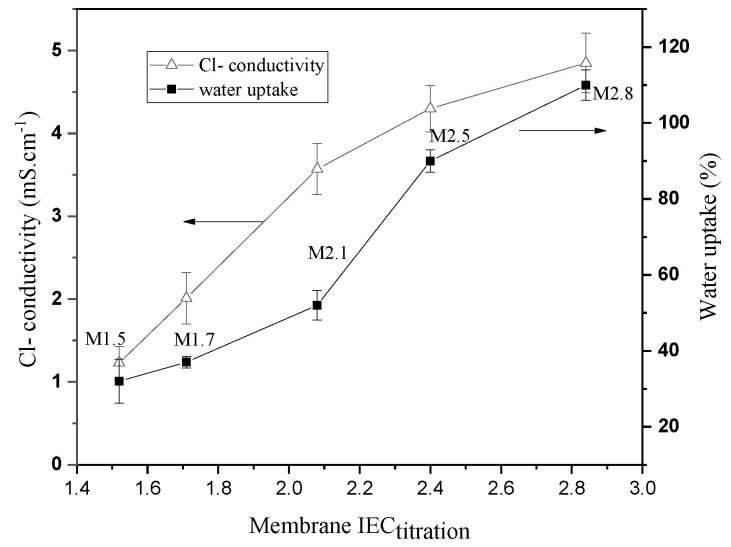
Water uptake and conductivity of the AEMs as function of titration *IEC* at 25 °C.

Similar to the water uptake, *IEC* has an impact on the conductivity of the membranes [54]. This conductivity is related to the number of charge carriers and their mobility, both of which are enhanced by *IEC*. Moreover, the charge carrier mobility increases with increasing water uptake as well as the connectivity between the ionic domains [55]. Figure 3 presents the conductivity of the membranes as function of their *IEC*s. It is interesting to note that the trend of the evolution of conductivity with *IEC* flattens at high *IEC*, thereby indicating a saturation effect. For membranes with an *IEC* between 1.5 and 2.1 mmol Cl^−^·g^−1^, the conductivity increases linearly with factor of more than 2.5 while above 2.1 mmol Cl^−^·g^−1^, the increase is much lower (less than 1.4) despite the much higher water uptake. Hence, for these high *IEC* membranes, where the presence of bulk water was evidenced, the impact of *IEC* on conductivity efficiency has a lower impact. The M2.5 membrane exhibits a behavior similar to that of the M2.8. Thus, the M2.5 membrane was not tested in the cell.

The commercial reference membrane FAA−3-50^®^ (thickness: 50 ± 5 µm; *IEC*: 1.85 mmol Cl^−^·g^−1^ polymer; *WU*: 17 wt.%) was found to have a Cl^−^ ion conductivity of 1.57 ± 0.2 mS·cm^−1^ in pure water at room temperature. By contrast, the conductivity value reported by the manufacturer is 3 to 8 mS·cm^−1^ (0.5 M NaCl at 25 °C, through-plane measuring cell), higher than that found in this study. The method of measuring and the presence of free salt could be the main reason for the variation in the membrane conductivity [56].

### 3.3. Cell Performance and Membrane Stability

#### 3.3.1. Charge/Discharge Tests

Four prepared AEMs were investigated in a well-established TMA-TEMPO/MV-based ORFB system. The test cells were primed at 20 mA·cm^−2^ for one charging/discharging cycle and afterwards aged for 100 cycles at 80 mA·cm^−2^. Finally, the capacity retention was probed at 20 mA·cm^−2^ by two additional charging/discharging cycles (Figures S5–S8). Table 4 presents the initial capacity of the cell measured at 20 mA·cm^−2^ and the capacity retained after 103 charging/discharging cycles. This value indicates the capacity fade due to the cross-contamination of the redox-active species through the membrane [28,57,58]. In addition, the capacity that is accessible at 80 mA·cm^−2^ is displayed. This value is typically lower since ohmic over-potentials limit the cell performance. It allows to evaluate the overall power capability of the membranes and provides an indication on their resistance. For each membrane type, three independent membrane samples were measured to avoid accidental error during membrane preparation. Membrane properties and cell performance of each independent sample are summarized in Table S1. For comparison, results of AEMs prepared in this study and a commercial reference membrane FAA−3-50^®^ are shown.

In this work, it is reasonable to neglect the impacts of electrolyte degradation on overall cell performance as the cycling stability of TMA-TEMPO/MV was evidenced in the literature [12]. Thus, the evaluation of the cell performance is focused on the selectivity and retention capability of the membranes. The capacity fade is exclusively related to the crossover of the redox-active species through the membrane, i.e., membrane ion selectivity. Using cyclic voltammetry, the crossover was evaluated.

**Figure 4:**
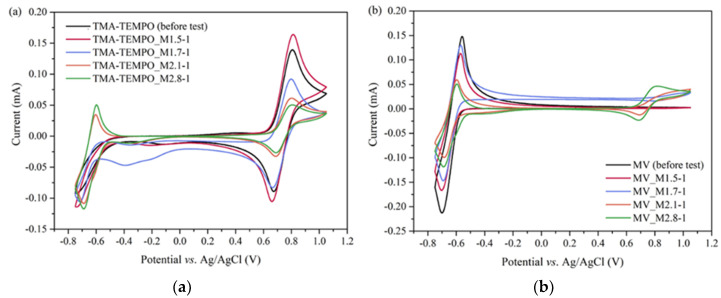
Cyclic voltammograms of TMA-TEMPO and MV solutions for each membrane before and after the cell cycling tests: (**a**) TMA-TEMPO half-cell and (**b**) MV half-cell. Scan rate: 200 mV·s^−1^.

According to Table 4, the capacity retention of the four AEMs after 103 charging cycles has a negative correlation with their ion selectivity, which is qualified by the degree of cross-contamination characterized via cyclic voltammetry. More precisely, from M1.5 to M2.8, the capacity retention decreased from 98.5 ± 0.5% to 29 ± 5%. Correspondingly, increased peak intensity of MV was observed in the recorded cyclic voltammograms of the electrolyte solutions of the TMA-TEMPO half-cell (Figure 4a). Similarly, increasing amount of TMA-TEMPO occurred in the MV half-cell of the battery at the end of the charge/discharge tests (Figure 4b). Only in the post-cycling analysis of the M1.5 cell no additional peaks emerged, indicating that neither MV nor TMA-TEMPO crossed the membrane. As for the other three membranes, they displayed different levels of capacity fade with respect to their *IEC*s and/or water uptake values. Compared to M1.7, both M2.1 and M2.8 display low-capacity retention regardless of the current applied, which mainly resulted from the irreversible high level of cross-contamination. This finding is in agreement with the distinctly higher water uptake of the two membranes and the presence of bulk water.

Moreover, the initial capacity associated with the different membranes was found to be different, especially membranes with high degree of crossover, revealed a relatively low initial capacity. This can be associated with crossover already taking place during the first charging cycle.

**Figure 5:**
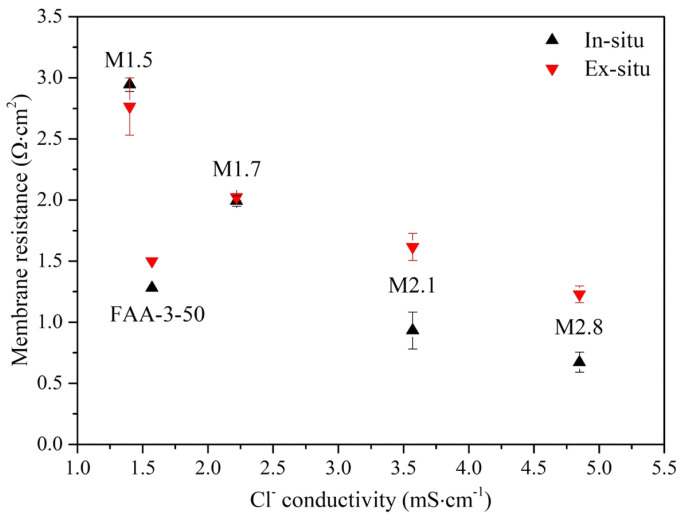
Membrane resistances compared to their chloride ion conductivity.

For practical applications, low cell resistance is essential for high current density operation [57] and a high-performance battery [5]. In this sense, charge/discharge cycling behavior was recorded at a relatively high current density of 80 mA·cm^−2^, when the cell resistance can have a large impact due to ohmic drop or ionic species diffusion limitation. To be more direct and comparative, all the cell resistances were corrected as membrane resistances by subtracting the cell resistance without a membrane. As expected, both of the ex-situ and in-situ membrane resistances declined with the increasing chloride ion conductivity and hence, the *IEC* value, as plotted in Figure 5. The deviation between the in-situ and ex-situ membrane resistances for the more conductive membranes i.e., M2.1 and M2.8 could be attributed to the different electrolyte media, the ex-situ membrane resistance was measured in water while the in-situ resistance was measured in the presence of redox-active species (aq). In general, both thickness and ionic conductivity of the membrane affect the membrane resistance, i.e., higher thickness and lower ionic conductivity lead to higher membrane resistance [58]. However, in the current work, as the thickness of the membranes does not vary notably, the *IEC* is the main parameter defining the membrane resistance. Even though the thickness increased from about 39 µm (M1.5) to about 60 µm (M2.8), the corresponding membrane resistance dramatically fell from 3 to 0.7 Ω·cm^2^. The commercial FAA-3-50^®^ membrane has 1.28 Ω·cm^2^ in-situ area resistance in the same conditions.

It becomes obvious that the membrane resistance has a strong influence on the overall cell performance, which reflects in the power capability of the test cells. When the current density is increased from 20 to 80 mA·cm^−2^, the capacity that remains accessible becomes increasingly limited as the ohmic drop gets higher. Indeed, the starting charge/discharge cell voltage, under current, gets higher/lower and the cell reaches the cutoff voltages earlier than those that have lower ohmic resistance, i.e., less time is left to charge/discharge.

**Figure 6:**
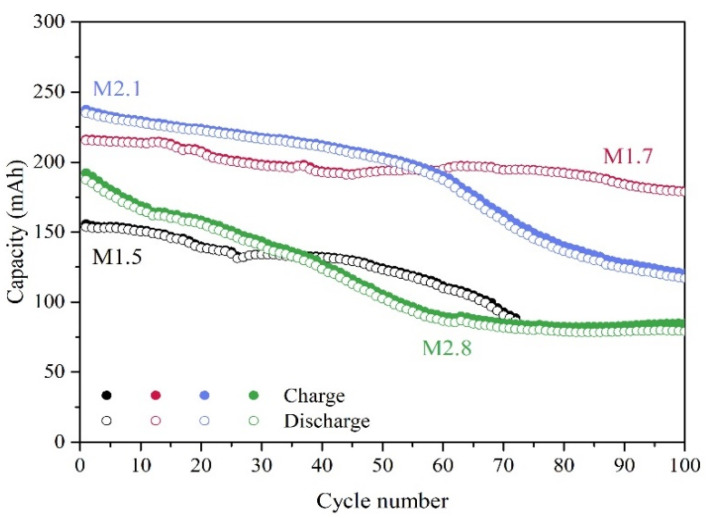
Charge/discharge capacity of the four kinds of PPOQ-DAPCl AEM at 80 mA·cm^−2^ over 100 cycles in TMA-TEMPO/MV (1.12 M/1.49 M) based ORFB single cell. The cell was primed at 20 mA·cm^−2^ for one cycle. Cutoff voltage: 0.9/1.5 V. The charge/discharge of the cell with M1.5 from 72nd cycle became invalid due to leakage; thus, data is not shown.

After priming at 20 mA·cm^−2^, all four AEMs were charged-discharged at 80 mA·cm^−2^ for 100 cycles, as shown in Figure 6. In the case of M1.5, the ohmic overpotential elevated dramatically due to its rather high membrane area resistance (2.945 ± 0.055 Ω·cm^2^), limiting the accessible capacity at 80 mA·cm^−2^ to 136 ± 18 mAh. M-1.5 that is exhibiting excellent capacity retention (98.5 ± 0.5) probed at 20 mA·cm^−2^ after 103 cycles. This result indicates that no crossover of TMA-TEMPO nor MV occurs as supported by CV measurements (Figure 4, red curves; Figure S9). However, the good retention comes at the price of limited power capability. Moreover, this failure to withdraw the full capacity is not irreversible capacity fade, instead, it will be available again when the charge/discharge current density drops back to 20 mA·cm^−2^. With lower membrane resistance than M1.5 and lower crossover than M2.1 or M2.8, M1.7 maintained a good balance between ion conductivity and selectivity of the membrane and, therefore, displayed the best capacity retention among the four membranes at 80 mA·cm^−2^.

As a reference, the commercial membrane FAA-3-50^®^ has nearly no crossover (Figure S13) like M1.5. Its low resistance; however, allows it to withdraw and store high capacity even at high current density (94%, 80 mA·cm^−2^). In fact, FAA-3-50^®^ has a chloride conductivity close to the conductivity of M1.5, with a water uptake even lower and a thickness 25% higher compared to M1.5. However, its membrane resistance is twice lower than that of M1.5 and is comparable with that of M2.1. The use of electrolyte solution, i.e., redox-active species dissolved in water, instead of pure water, seems to influence the conductive properties of the membrane, with evolution being beneficial for FAA-3-50^®^.

**Figure 7:**
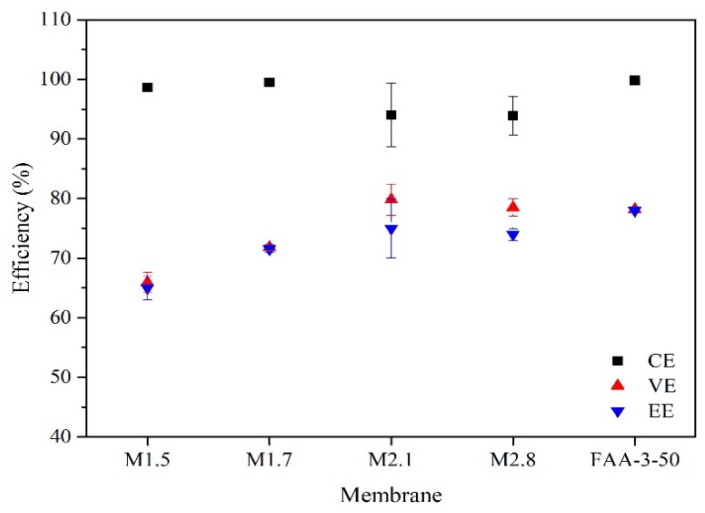
CE, VE and EE of the prepared AEMs and commercial membrane FAA-3-50^®^ operated at 80 mA·cm^−2^ for 100 cycles.

Coulombic efficiency (CE) and voltage efficiency (VE) are two additional parameters to evaluate the performance and reversibility of a battery system. As shown in Figure 7, the M1.5-based battery, using a membrane with high active material retention, exhibited an average CE of 99.1 ± 0.1%. Likewise, with the cell employing M1.7, a membrane with slight crossover, it was found to be above 99%. On the other hand, M2.1 and M2.8 displayed CE of 94.0 ± 5.3% and 93.9 ± 3.2%, respectively, which is an indication of the strong crossover of active species [5]. VE, on the other hand, is an indication of the internal resistance of the cell [59]. The test cells with M2.1 and M2.8, as expected, showed the highest VE (around 80% at 80 mA·cm^−2^) due to their low associated cell resistances. In contrast, the cell employing M1.5, membrane with highest resistance, showed the lowest VE (65.9 ± 1.7%, Figure 7) among the tested membranes.

Energy efficiency (EE) indicates the energy loss of a battery system. It is typically between 50 to 90% for RFBs depending on the material properties of the membrane, the active species used and applied current density [5]. Among the four tested membranes, M2.1 and M2.8 have the highest EE due to their higher VE (lower resistance) followed by M1.7 (Figure 7). The cell with M1.7 revealed an excellent CE of above 99% as that of FAA-3-50^®^ does. However, its relatively high membrane resistance limited its EE. The same holds true for membrane M1.5.

#### 3.3.2. Polarization Curve

**Figure 8:**
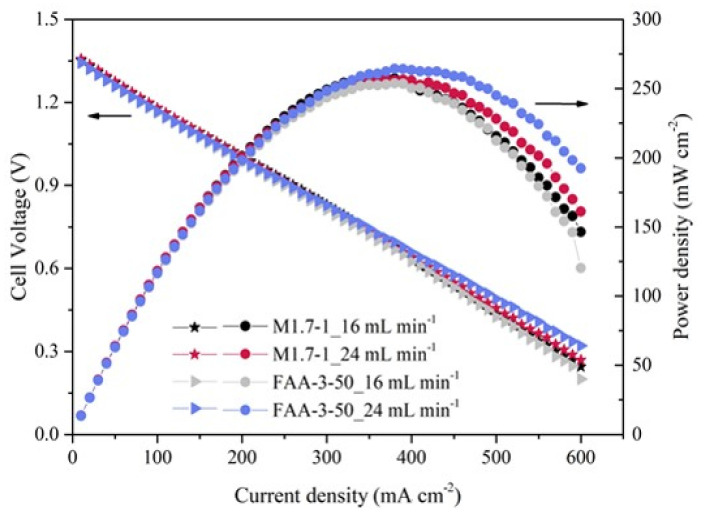
Discharge polarization curves and power densities of M1.7-1 and FAA-3-50^®^ membranes over current density.

With the best overall cell performance among the four AEMs, the discharge polarization curve of M1.7-1 in a single cell at 100% SOC was recorded in an effort to reveal the performance loss and evaluate its suitably for practical flow battery application. The cell was operated within the range of 10 to 600 mA·cm^−2^ directly after the charge/discharge test, with the polarization curve of FAA-3-50^®^ for comparison. As shown in Figure 8, the M1.7-1-based battery exhibited slightly higher peak power density than the commercial reference FAA-3-50^®^ (258 vs. 254 mW·cm^2^) at about 360 mA·cm^−2^ with a standard flow rate of 16 mL·min^−1^. The linear relation of voltage and current density at low-current densities suggests low activation over-potentials at the electrodes [60]. The cell voltages of M1.7 and FAA-3-50^®^ both started to deviate slightly from the linear trend at current densities above 350 mA·cm^−2^, which suggested the gradual shift from loss caused by internal resistance to that by mass transport of the redox-active molecules. Therefore, the electrolyte flow rate was elevated to 24 mL·min^−1^ in order to investigate the influence of mass transport. Increasing the flow rate showed minor effects on the polarization behavior of the M1.7-based cell. While the maximum power density at 16 and 24 mL·min^−1^ revealed similar values (258 and 257 mW·cm^2^), the trend of the polarization curve becomes fully linear. It follows Ohm’s law well. Hence, the performance of the test cell is dominated by its internal resistance and the resulting ohmic over-potentials. As these values mainly originate in the membrane resistance, effects of mass transport can be neglected. A test cell employing the reference membrane FAA-3-50^®^ displayed a 4% increase of retrievable power density at current densities above 350 mA·cm^−2^ and a deviation from linear voltage-current behavior. In this setup, the depletion of redox-active species is compensated by an increased electrolyte flow. However, as both test cells are dominantly defined by their membrane resistance, future research needs to focus on the preparation of AEMs with high chloride ion conductivity to further increase the retrievable power.

#### 3.3.3. Membrane Durability

**Figure 9:**
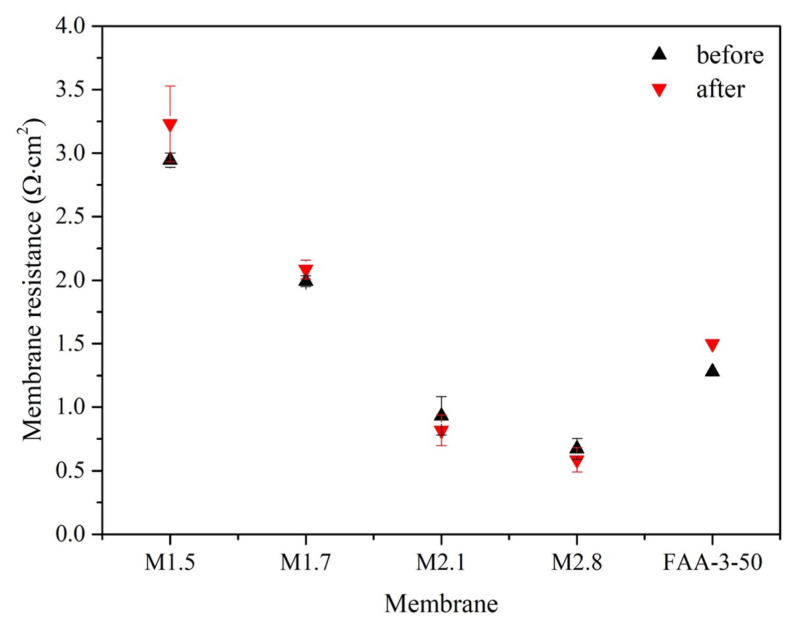
Membrane resistance of the four prepared AEMs and commercial membrane FAA-3-50^®^ before and after the charge/discharge cycling tests.

To assess the long-term durability of the AEMs, the membrane resistances after the cell tests were recorded in comparison to those before cell tests. As can be seen in Figure 9, the difference in the membrane resistance is small and remains within the error margin. This indicates the absence of membrane aging due to effects like a reduction of the *IEC* (due to ionic species loss), which would have caused an increase in resistance.

After cell disassembly, no observable breaks or holes appeared in all tested membranes. Morphology changes were further checked under the microscope. The mechanical deformation, which is visible on the surface of the membranes, is caused by the pressure applied on the membrane upon assembling the cell (Figure S14). The micrographs support the results found in the resistance measurement and suggest a good mechanical and chemical stability of the employed AEMs, benefiting from the noncorrosive nature of neutral electrolytes and the high chemical stability of the QA cations.

## 4. Conclusions

In this study, five AEMs based on PPO grafted with various amounts of poly (DAPCl) cations were fabricated via a rapid UV-irradiation method and four of them were tested in a TMA-TEMPO/MV-based AORFB. Membranes with high *IEC* lead to higher water uptake, higher chloride mobility and lower membrane resistance. However, the large water uptake is detrimental for the cyclability as active species crossover can be observed during cycling. Membranes with a low *IEC* and/or water uptake have been found to display low TMA-TEMPO/MV crossover and good capacity retention after 100 consecutive charging/discharging cycles. The M1.7 membranes with moderate membrane resistance and low crossover were found to display the best overall cell performance among the four tested membranes at high current density of 80 mA·cm^−2^, with highest capacity retention of 83.5 ± 0.5%, excellent CE of 99.5 ± 0.1% and moderate EE of 71.5 ± 0.5%. In this case, the *IEC* of M1.7 keeps a good balance of the chloride ion conductivity (2.22 ± 0.08 mS·cm^−1^) over water uptake (37 ± 1%), which induces low membrane resistance and crossover. When all the results are comprehensively considered, the M1.7 membrane appears to have the highest applicability for AORFB because of its high power density and good retention capacity. Our results show a promising understanding of the correlations between the different membrane properties and their corresponding cell performance and stability. Therefore, further research efforts should be focused on preparing a more conductive or less resistant AEM with reduced electrolyte crossover, increased energy efficiency and increased power density.

## Figures and Tables

**Table 1:** Monomer to polymer ratio and theoretical IEC of the membranes.

Membrane	Feed DAPCl to PPO-Q Molar Ratio	Theoretical IEC (mmol Cl^−^·g^−1^ Polymer)
M1.5	1.4	1.96
M1.7	2	2.23
M2.1	3	2.58
M2.5	4	2.85
M2.8	6	3.3

**Table 2:** Chemical composition and membrane properties of the five prepared AEMs.

Membrane	Thickness (µm)	Titration DAPCl to PPO-Q Molar Ratio	DAPCl Conversion (%) *	Titration *IEC* (mmol Cl^−^·g^−1^ Polymer)
M1.5	39 ± 3	0.65	46	1.52 ± 0.12
M1.7	45 ± 3	0.95	48	1.71 ± 0.11
M2.1	57 ± 3	1.7	56	2.08 ± 0.05
M2.5	58 ± 3	2.5	62	2.40 ± 0.07
M2.8	60 ± 3	4	66	2.84 ± 0.07

* $\mathrm{DAPCl}\;\mathrm{conversion}\;\left(\%\right)=\frac{\mathrm{DAPCl}\;\mathrm{to}\;\mathrm{PPO}-Q\;\mathrm{titration}}{\mathrm{DAPCl}\;\mathrm{to}\;\mathrm{PPO}-Q\;\mathrm{feed}}\times 100$.

**Table 3:** Amount of freezable and non-freezable water in the membranes, their enthalpy and melting temperature.

Membrane	Water Hydration (λ)	Δ*H*_f_ DSC (J·g^−1^ Wet Sample) ^a^	Δ*H*_f_ DSC (J·g^−1^ Water) ^b^	Freezable Water (%) = Δ*H*_f_,_DSC_/Δ*H*_f_, Pure Water ^c^	Freezable λ in the Membrane ^d^	Non-Freezable λ in the Membrane	T_m_ (K) ^e^
M1.5	11.6 ± 1.4	0	0	0	0	11.6 ± 1.4	-
M1.7	11.8 ± 0.3	8 ± 3.1	30 ± 11	9 ± 3.5	0.4 ± 0.2	11.4 ± 0.2	255
M2.1	14.2 ± 0.7	15 ± 1.7	45 ± 5	14 ± 1.5	1 ± 0.1	13 ± 0.1	268
M2.8	21.5 ± 0.3	52 ± 6	98 ± 11	29 ± 3.3	7 ± 0.8	14.5 ± 0.8	273

^a:^ Obtained from the DSC. ^b:^ Δ*H*_f_ DSC (J·g^−1^ water) = (Δ*H*_f_ DSC, J·g^−1^ wet membrane) × (sample weight, g)/(water weight in the sample, g). ^c:^ Melting enthalpy of pure water is taken as 334 J·g^−1^. ^d:^ Freezable λ in the membrane was calculated using Equation (5). ^e:^ T_m_ (K) is the melting temperature of the water absorbed in the membrane.

**Table 4:** Summary of initial capacity and capacity retention of the prepared AEMs and commercial membrane FAA-3-50^®^ at 20 and 80 mA·cm^−2^.

Membrane	Capacity 1st Cycle at 20 mA·cm^−2^ [mAh]	Accessible Capacity at 80 mA·cm^−2^ [mAh, Retention (%) ^a^]	Capacity 103rd Cycle at 20 mA·cm^−2^ [mAh, Retention (%) ^b^]
M1.5	283 ± 48	136 ± 18 (41 ± 4)	279 ± 48 (98.5 ± 0.5)
M1.7	297 ± 3	207 ± 8 (83.5 ± 0.5)	261 ± 2 (88 ± 0)
M2.1	218 ± 39	174 ± 45 (55 ± 16)	107 ± 24 (49 ± 4)
M2.8	216 ± 33	174 ± 22 (34 ± 8)	59 ± 8 (29 ± 5)
FAA-3-50^®^	301	244 (94)	288 (95)

^a^: Calculated by dividing the discharge capacity of 101st cycle by that of 2nd cycle. ^b^: Calculated by dividing the discharge capacity of 103rd cycle by that of 1st cycle.

## Data Availability

The raw data and samples presented in this study are available on request from the corresponding authors. All relevant processed data is shown in the manuscript and supplementary material associated.

## Supplementary Materials

The following are available online at https://www.mdpi.com/article/10.3390/membranes11050367/s1, Figure S1. Homemade membranes ion conductivity measuring cell, Figure S2. ^1^H NMR spectrum of PPO-Br (a) in CDCl_3_ and PPO-Q (b) in DMSO-d_6_, Figure S3. 1H NMR (400 MHz) spectra of N-allylpiperidine in CDCl3 (i) and of N,N-diallylpiperidinium chloride (DAPCl) in H2O (ii), Figure 4. TGA curves of PPO, PPO-Br, PPO-Q, DAPCl and M2.8 measured under N_2_ at 10 K·min^−1^, Figure S5. Charge/discharge capacity and coulombic efficiency of M1.5 in TMA-TEMPO/MV based ORFBs at room temperature. Catholyte: 1.12 M TMA-TEMPO (aq.). Anolyte: 1.49 M MV (aq.), Figure S6. Charge/discharge capacity and coulombic efficiency of M1.7 in TMA-TEMPO/MV based ORFBs at room temperature. Catholyte: 1.12 M TMA-TEMPO (aq.). Anolyte: 1.49 M MV (aq.), Figure S7. Charge/discharge capacity and coulombic efficiency of M2.1 in TMA-TEMPO/MV based ORFBs at room temperature. Catholyte: 1.12 M TMA-TEMPO (aq.). Anolyte: 1.49 M MV (aq.), Figure S8. Charge/discharge capacity and coulombic efficiency of M2.8 in TMA-TEMPO/MV based ORFBs at room temperature. Catholyte: 1.12 M TMA-TEMPO (aq.). Anolyte: 1.49 M MV (aq.), Figure S9. Cyclic voltammograms of TMA-TEMPO and MV solutions (100 µL in 4.6 mL 0.5 M NaCl (aq.)) for M1.5 before and after the cell cycling tests. (a) TMA-TEMPO. (b): MV. Scan rate: 200 mV·s^−1^. Background subtracted before plotting; the fourth scan was chosen to prepare the comparative graphs, Figure S10. Cyclic voltammograms of TMA-TEMPO and MV solutions (100 µL in 4.6 mL 0.5 M NaCl (aq.)) for M1.7 before and after the cell cycling tests. (a): TMA-TEMPO. (b): MV. Scan rate: 200 mV·s^−1^. Background subtracted before plotting; the fourth scan was used to prepare the comparative graphs, Figure S11. Cyclic voltammograms of TMA-TEMPO and MV solutions (100 µL in 4.6 mL 0.5 M NaCl (aq.)) for M2.1 before and after the cell cycling tests. (a): TMA-TEMPO. (b): MV. Scan rate: 200 mV·s^−1^. Background subtracted before plotting; the fourth scan was chosen to prepare the comparative graphs, Figure S12. Cyclic voltammograms of TMA-TEMPO and MV solutions (100 µL in 4.6 mL 0.5 M NaCl (aq.)) for M2.8 before and after the cell cycling tests. (a): TMA-TEMPO. (b): MV. Scan rate: 200 mV·s^−1^. Background subtracted before plotting; the fourth scan was used to prepare the comparative graphs, Figure S13. Cyclic voltammograms of TMA-TEMPO and MV solutions (100 µL in 4.6 mL 0.5 M NaCl (aq.)) for FAA-3-50^®^ after the cell cycling test. (a): TMA-TEMPO. (b): MV. Scan rate: 200 mV·s^−1^. Background subtracted before plotting; the fourth scan was chosen to prepare the comparative graphs, Figure S14. Micrographs of the AEMs before and after cell tests in wet form: (a) M1.5-3 before, (b) M1.5-3 after, (c) M1.7-2 before, (d) M1.7-2 after, (e) M2.1-2 before, (f) M2.1-2 after, (g) M2.8-3 before and (h) M2.8-3 after. Bar size: 200 µm, Table S1. Summary of the membrane properties and cell performances of all tested AEMs.
