# Supplementary material for: Study of Anion Exchange Membrane Properties Incorporating N-spirocyclic Quaternary Ammonium Cations and Aqueous Organic Redox Flow Battery Performance
Source: Membranes (Basel). 2021 May 18;11(5):367. doi: 10.3390/membranes11050367 (PMC8158339; doi:10.3390/membranes11050367)
Supplement: Supplementary file 1 [file membranes-11-00367-s001.zip › membranes-1230106-supplementary.pdf]

membranes

Supplementary Materials

Study of Anion Exchange Membrane Properties Incorporating *N*-spirocyclic Quaternary Ammonium Cations and Aqueous Organic Redox Flow Battery Performance

Misgina Tilahun Tsehaye[a,†], Xian Yang[b,c,d,†], Tobias Janoschka[d] *, Martin D Hager[b,c], Ulrich S. Schubert[b,c], Fannie Alloin[a,e] *, Cristina Iojoiu[a,e] *

[a] Univ. Grenoble Alpes, Univ. Savoie Mont Blanc, CNRS, Grenoble INP, LEPMI, 38 000 Grenoble, France
[b] Laboratory of Organic and Macromolecular Chemistry (IOMC), Friedrich Schiller University Jena, Humboldtstrasse 10, 07743 Jena, Germany
[c] Center for Energy and Environmental Chemistry Jena (CEEC Jena), Friedrich Schiller University Jena, Philosophenweg 7a, 07743 Jena, Germany
[d] JenaBatteries GmbH, Otto-Schott-Strasse 15, 07745 Jena, Germany
[e] Réseau sur le Stockage Electrochimique de l'Energie (RS2E), CNRS, FR3459, 80 039 Amiens Cedex, France
* Correspondence: Cristina.iojoiu@lepmi.grenoble-inp.fr, Fannie.Alloin@lepmi.grenoble-inp.fr, tobias.janoschka@jenabatteries.de
† Authors contributed equally.

Citation: Tsehaye, M.T.; Yang, X.; Janoschka, T.; Hager, M.D.; Schubert, U.S.; Alloin, F.; Iojoiu, C. Study of Anion Exchange Membrane Properties Incorporating *N*-spirocyclic Quaternary Ammonium Cations and Aqueous Organic Redox Flow Battery Performance. *Membranes* **2021**, *11*, 367. https://doi.org/10.3390/membranes11050367

Academic Editor: Patric Jannasch

Received: 05 May 2021
Accepted: 14 May 2021
Published: 18 May 2021

Publisher's Note: MDPI stays neutral with regard to jurisdictional claims in published maps and institutional affiliations.

Copyright: © 2021 by the authors. Licensee MDPI, Basel, Switzerland. This article is an open access article distributed under the terms and conditions of the Creative Commons Attribution (CC BY) license (http://creativecommons.org/licenses/by/4.0/).

SUMMARY

Number of pages: 10
Number of Figures: 14
Number of Tables: 1

Conductivity Measuring Cell

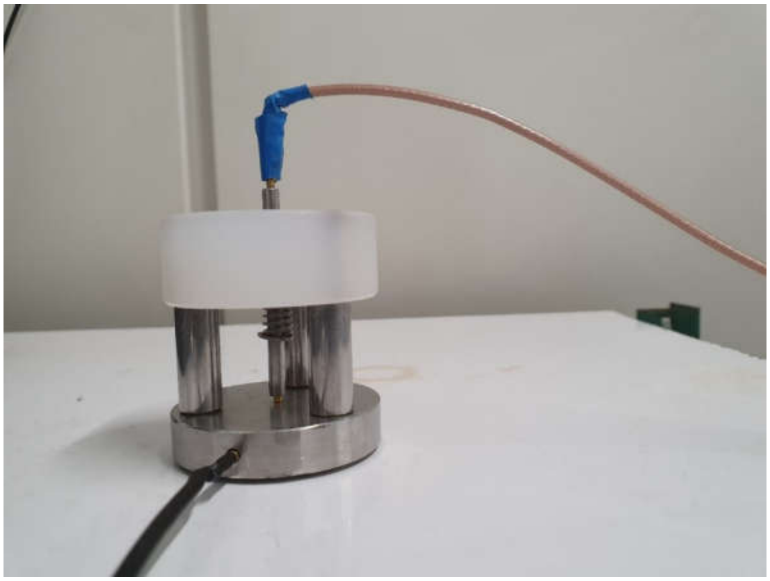

Figure S1. Homemade membranes ion conductivity measuring cell.

Analytical data

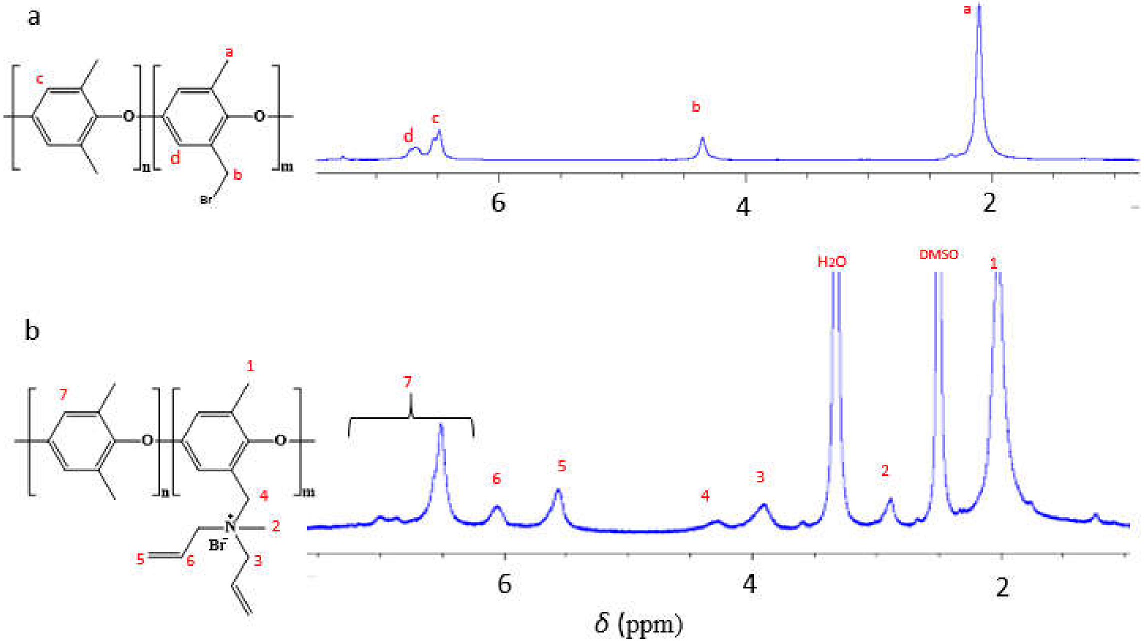

Figure S2. ^{1}H NMR spectrum of PPO-Br (**a**) in $CDCl_3$ and PPO-Q (**b**) in DMSO-d_6.

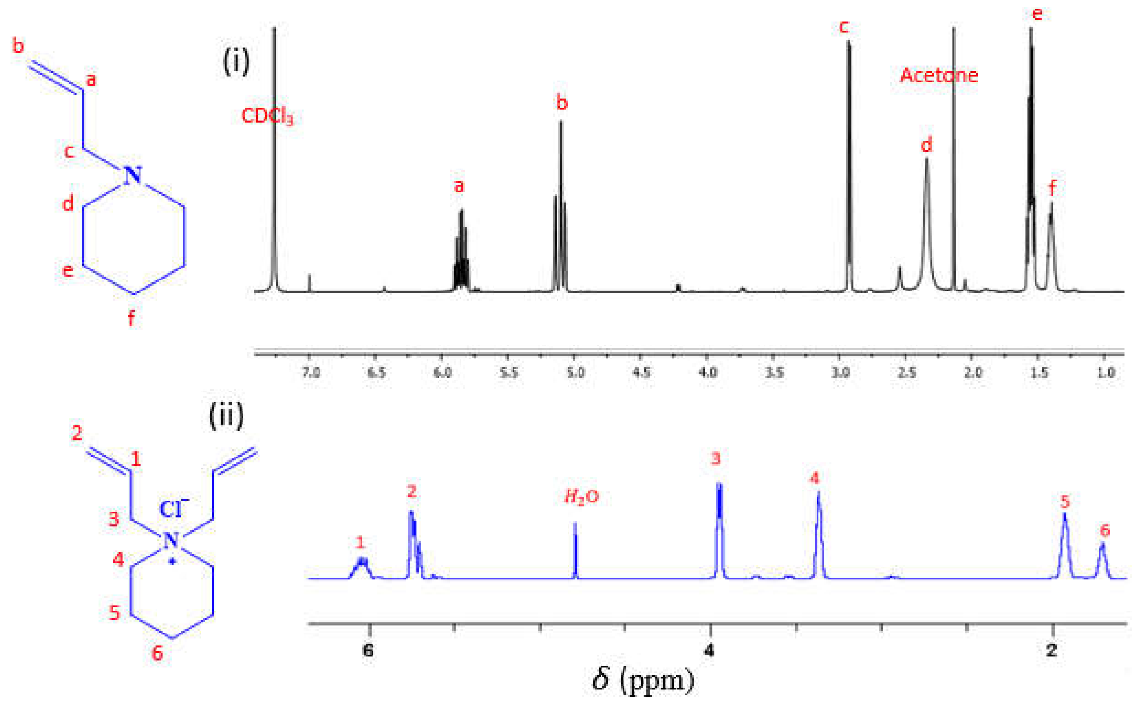

Figure S3. ^1H NMR (400 MHz) spectra of *N*-allylpiperidine in $CDCl_3$ (i) and of *N*,*N*-diallylpiperidinium chloride (DAPCl) in H_2O (ii).

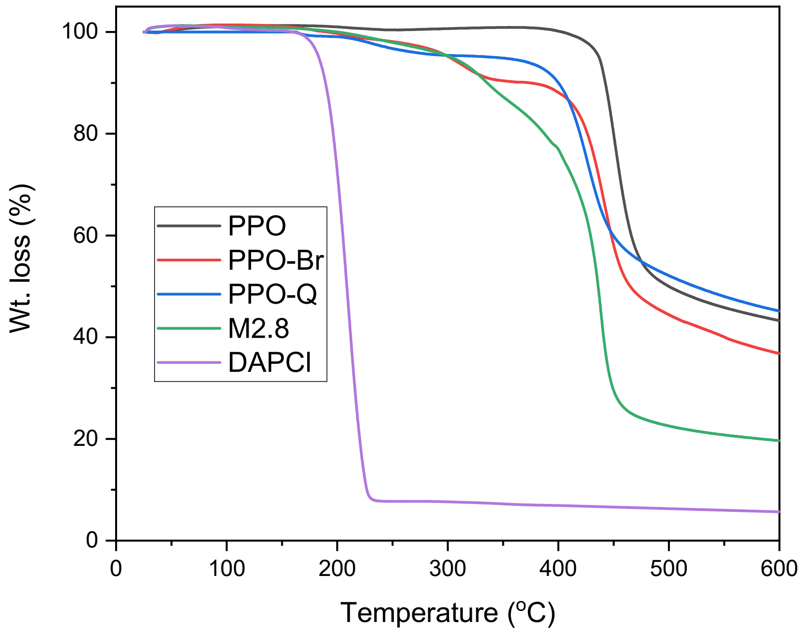

Figure 4. TGA curves of PPO, PPO-Br, PPO-Q, DAPCl and M2.8 measured under N_2 at 10 K·min^{-1}.

Membrane properties

Table S1. Summary of the membrane properties and cell performances of all tested AEMs.

Membrane	IEC	Thickness (μm)	Water uptake (%)	Cl^- conductivity ($mS\cdot cm^{-1}$)	Membrane resistance (before/after, $\Omega\cdot cm^2$)	CE (%)	EE (%)	Capacity (1st cycle, mAh at 20 $mA\cdot cm^2$)	Accessible capacity (mAh, retention (%) at 80 $mA\cdot cm^2$)	Capacity (103rd cycle, mAh, retention (%) at 20 $mA\cdot cm^2$)
M1.5-1	1.58	39	30	1.30	3.000/3.630	99.0 [a]	63 [a]	331	119 (37)	327 (99)
M1.5-2	1.64	38	34	1.50	2.890/3.100	99.2 [a]	67 [a]	235	154 (45)	231 (98)
M1.5-3[b]	1.36	39	20	0.90	4.275/5.170	-	-	300	- (-)	-
M1.7-1	1.84	46	38	2.30	1.950/2.160	99.4	71	294	199 (84)	259 (88)
M1.7-2[c]	1.56	46	34	1.58	1.005/1.405	98.9	74	286	236 (31)	214 (75)
M1.7-3	1.72	44	36	2.15	2.035/2.010	99.6	72	300	215 (83)	263 (88)
M2.1-1	2.02	51	52	3.42	0.945/0.760	86.5	70	214	137 (77)	113 (53)
M2.1-2	1.98	58	48	3.30	1.110/0.985	97.1	74	173	148 (38)	76 (44)
M2.1-3	2.10	64	57	4.00	0.740/0.710	98.4	81	266	237 (49)	133 (50)
M2.8-1	2.78	56	106	4.42	0.730/x	96.5	75	255	190 (42)	69 (31)
M2.8-2	2.94	60	115	5.30	0.555/0.490	89.4	72	219	189 (23)	49 (22)
M2.8-3	2.81	62	110	4.82	0.730/0.680	95.8	74	174	142 (38)	60 (34)

x: Not acquired. Hence, the average membrane resistance ($0.585 \pm 0.095\ \Omega\cdot cm^2$) of M2.8 after the cell cycling test was calculated from M2.8-2 and M2.8-3.

[a]: Invalid data points, where capacities turned zero amid cycling, were removed from calculation of CE and EE of M1.5-1 and M1.5-2 (see Figure S4).

[b]: M1.5-3 was left out when calculating the M1.5 average values because it failed to charge and discharge at 80 $mA\cdot cm^{-2}$ due to its rather high membrane resistance.

[c]: M1.7-2 was left out when calculating the M1.7 average values because its membrane properties and cell performance deviated distinctly from the other two samples.

Charge/discharge behavior

Figures S5-S8 show the charge-discharge behavior of the four AEMs. All membranes were tested in the same TMA-TEMPO/MV based ORFB and experienced the same electrochemical procedure. The first charge-discharge was performed at low current density of 20 mA·cm^{-2}. Subsequently, they were cycled at high current density of 80 mA·cm^{-2} for 100 times. Finally, they were charge-discharged again at 20 mA·cm^{-2} twice. In this way, it is possible to qualify for each membrane type the influence of membrane resistance (or conductivity) and membrane selectivity on the overall capacity fade.

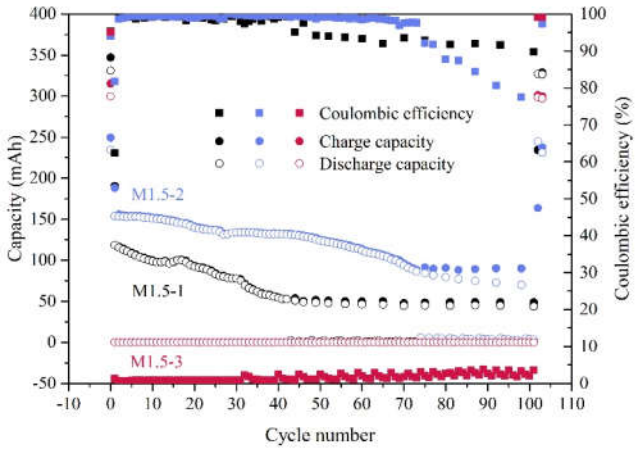

Figure S5. Charge/discharge capacity and coulombic efficiency of M1.5 in TMA-TEMPO/MV based ORFBs at room temperature. Catholyte: 1.12 M TMA-TEMPO (aq.). Anolyte: 1.49 M MV (aq.).

The charge/discharge capacity of M1.5-1 vary slightly due to a temperature effect. All cells were operated in an air-conditioned room with a constant room temperature of 22 °C when without interference. However, during daytime at working hours, the room door is inevitably opened and closed. Hence, the actual room temperature is higher than 22 °C. After work, the room is left undisturbed. The room temperature holds back stable at 22 °C. Higher temperature reduces the cell resistance and makes the active species in solution move faster, resulting in higher capacity. As can be seen from Figure S5, in the beginning, M1.5-1 has a smooth curve of capacity decay because it was off-work hours. Then it went to working hours, the apparent capacity increased due to the elevated room temperature. Afterwards, it faded again faster and kept the similar capacity decay rate as in the beginning. The trend went on until it reached 45th cycle. Afterwards, the capacity oscillated within zero and 50 mAh. This probably attributes to the increased resistance (Table 1, 3.000/3.630 Ω·cm^2), which is around the edge of the threshold resistance that enables the cell to charge/discharge at high current density. Similar oscillation of capacity was observed for M1.5-2 that also had a high membrane resistance of 2.890 Ω·cm^2 and increased resistance after 100 cycles.

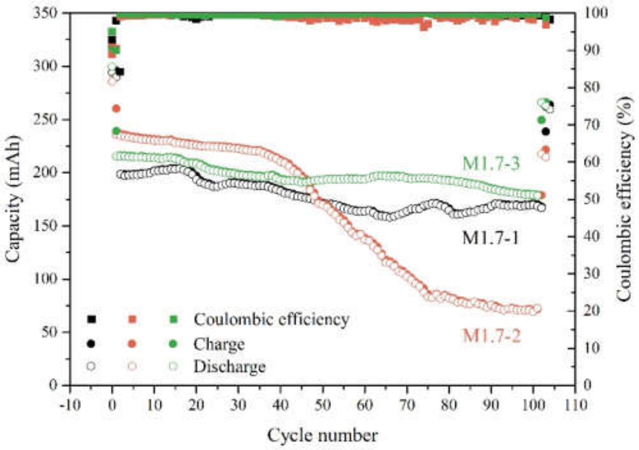

Figure S6. Charge/discharge capacity and coulombic efficiency of M1.7 in TMA-TEMPO/MV based ORFBs at room temperature. Catholyte: 1.12 M TMA-TEMPO (aq.). Anolyte: 1.49 M MV (aq.).

Similarly, the charge/discharge capacity curve of M1.7-1 and M1.7-3 displayed strong temperature effect. M1.7-2, however, had abnormal behavior. It has a Cl^- conductivity of 1.58 $mS{\cdot}cm^{-1}$, which is lower than M1.7-1 (2.30 $mS{\cdot}cm^{-1}$) and M1.7-3 (2.15 $mS{\cdot}cm^{-1}$). Hence, its corresponding membrane resistance is expected to be higher than the other two. Surprisingly, its membrane resistance is much lower, half the value of M1.7-1 and M1.7-3. In the beginning, the capacity of M1.7-2 faded slowly. From 35th cycle, the capacity decay rate suddenly sped up until at 75th cycle, when it went back to normal speed. From the cyclic voltammograms of the electrolyte solutions investigated post cycling (Figure S10, blue curve) it can be concluded that small amounts of cross-contamination of TMA-TEMPO and MV occurred in the case of M1.7-2. Nevertheless, it cannot cause such an abrupt speed-up if electrolyte leakage is not counted. The most probable explanation could be the gradual increase in cell resistance during the cycling process. When the cell resistance finally stopped increasing, the capacity decay rate went back to its original level. The reason for the abrupt change of cell resistance is yet unclear; however, its membrane properties do have apparent deviation from the other two membrane samples (M1.7-1 and M1.7-3). The strange behavior of M1.7-2 was taken as an independent case where the problem probably relates to the preparation of this membrane sample. As a result, M1.7-2 was removed from the calculation of the average value.

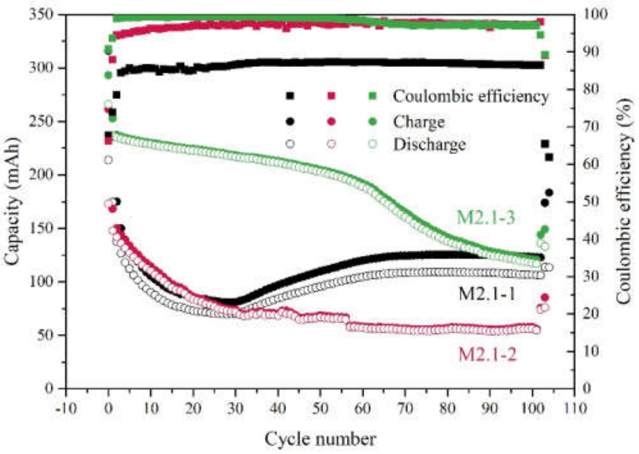

Figure S7. Charge/discharge capacity and coulombic efficiency of M2.1 in TMA-TEMPO/MV based ORFBs at room temperature. Catholyte: 1.12 M TMA-TEMPO (aq.). Anolyte: 1.49 M MV (aq.).

The charge/discharge capacity of M2.1-1 exhibited unusual increase starting from 30th cycle and gradually reached a plateau. The final capacity of M2.1-1 at 80 mA·cm^{-2} resembled that of M2.1-3, which had similar membrane resistance (Table 1) and degree of crossover (Figure S11, yellow and green curves) after cycling test. The abnormal capacity valley of M2.1-1 could be an independent case, where it took longer time for the membrane to be well integrated into the mass transport process (*e.g.*, swelling of the membrane).

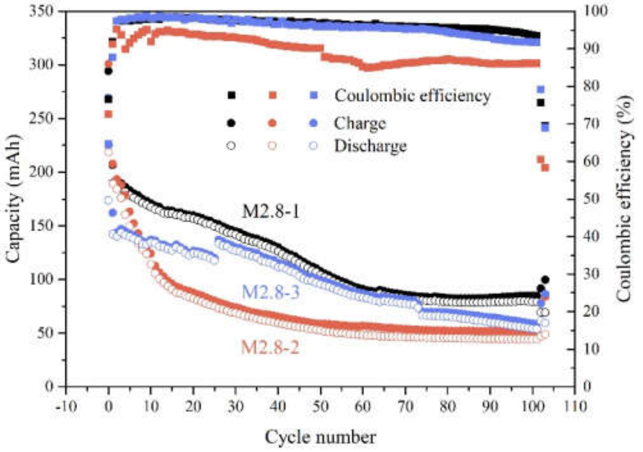

Figure S8. Charge/discharge capacity and coulombic efficiency of M2.8 in TMA-TEMPO/MV based ORFBs at room temperature. Catholyte: 1.12 M TMA-TEMPO (aq.). Anolyte: 1.49 M MV (aq.).

In general, the three tested M2.8 membranes share the similar trend in capacity fade, *i.e.* rather fast loss in capacity right from the start and then gradually reach an equilibrium. That is when the crossover of the catholyte and anolyte (Figure S12) to the extent that the electrolyte composition on both sides is almost the same. The abrupt elevation of capacity with M2.8-3 at around 25th cycle could be due to the accumulative droplets attached to the reservoir wall fell into the bulk solution. Likewise, at around 73th cycle, small amount of droplets distributed loosely on the reservoir wall, thus failing to flow back to the bulk solution, resulting in discontinuous capacity decay.

Crossover of active material (TMA-TEMPO and MV)

Cyclic voltammetry was used as a tool to qualify the cross-contamination of the electrolytes after cell cycling test for each membrane. Electrolyte solutions of TMA-TEMPO and MV both before and after the cell tests were measured under the same condition. In principle, the degree of cross-contamination can be quantified through comparison of the redox peak intensities. In practice, however, the concentrations of the starting electrolyte solutions are unequal, which implies inevitably osmosis-induced water transfer during the cycling experiments. It means that the concentrations of TMA-TEMPO/MV solutions will change accordingly in some degree. For example, in Figure S9, the redox peak intensities of TMA-TEMPO with the three M1.5 membranes are stronger than that before cell test, whereas in MV side, the trend reversed. This is because water was driven from TMA-TEMPO side to MV side by the osmosis pressure originated from the concentration difference. However, one can still tell from the curve whether crossover of electrolyte happens or not and to how large extent the crossover is. As a result, no crossover occurred for FAA-3-50® and M1.5, small crossover for M1.7, middle crossover for M2.1 and strong crossover for M2.8.

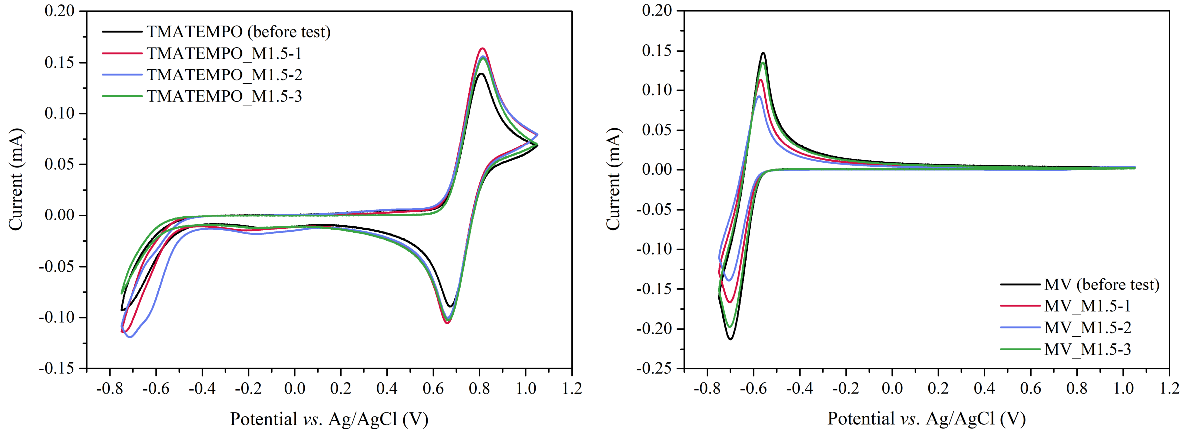

(**a**) (**b**)

Figure S9. Cyclic voltammograms of TMA-TEMPO and MV solutions (100 μL in 4.6 mL 0.5 M NaCl (aq.)) for M1.5 before and after the cell cycling tests. (**a**) TMA-TEMPO. (**b**): MV. Scan rate: 200 mV·s^{-1}. Background subtracted before plotting; the fourth scan was chosen to prepare the comparative graphs.

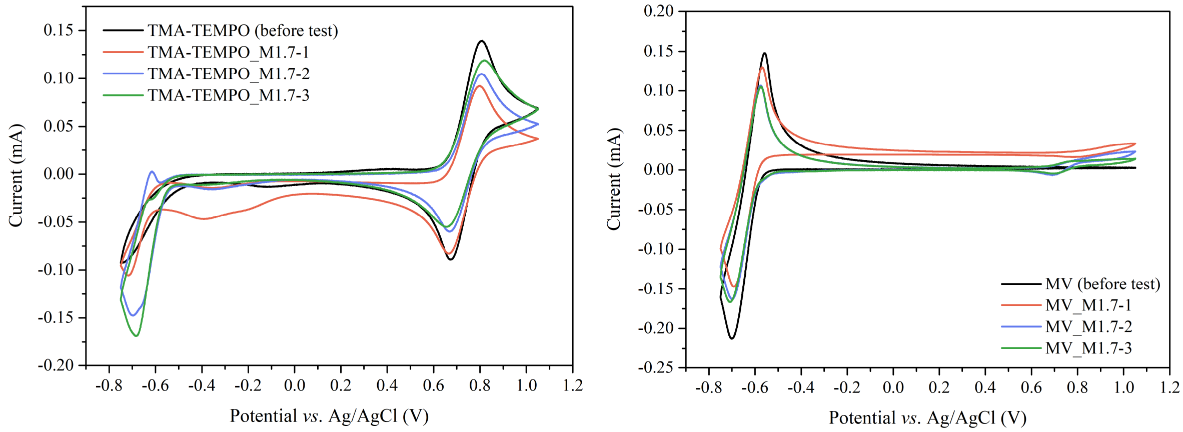

(**a**) (**b**)

Figure S10. Cyclic voltammograms of TMA-TEMPO and MV solutions (100 μL in 4.6 mL 0.5 M NaCl (aq.)) for M1.7 before and after the cell cycling tests. (**a**): TMA-TEMPO. (**b**): MV. Scan rate: 200 mV·s^{-1}. Background subtracted before plotting; the fourth scan was used to prepare the comparative graphs.

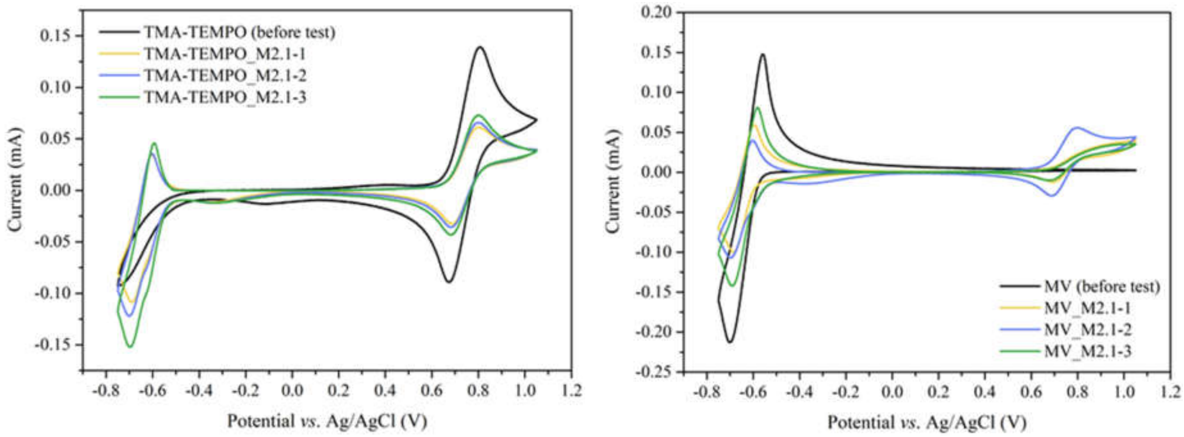

(**a**) (**b**)

Figure S11. Cyclic voltammograms of TMA-TEMPO and MV solutions (100 µL in 4.6 mL 0.5 M NaCl (aq.)) for M2.1 before and after the cell cycling tests. (**a**): TMA-TEMPO. (**b**): MV. Scan rate: 200 mV·s-1. Background subtracted before plotting; the fourth scan was chosen to prepare the comparative graphs.

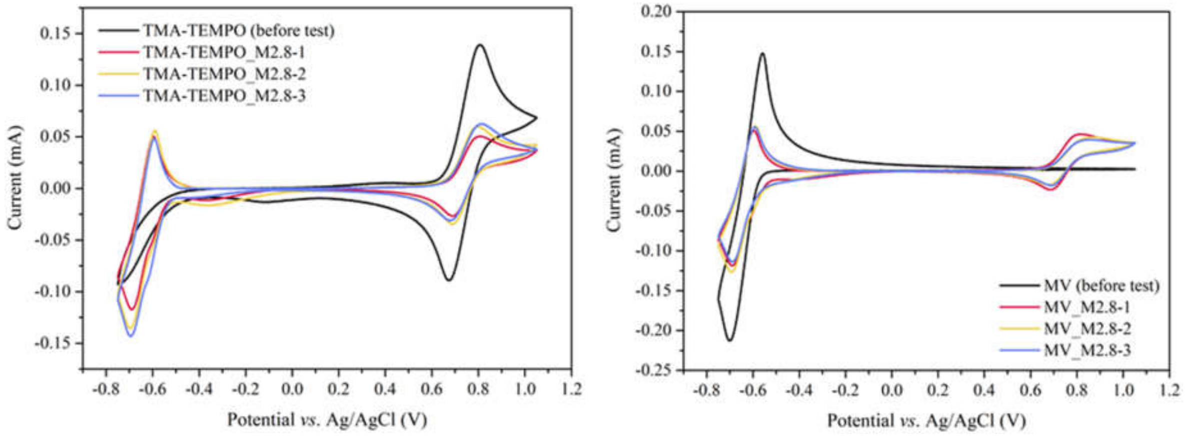

(**a**) (**b**)

Figure S12. Cyclic voltammograms of TMA-TEMPO and MV solutions (100 µL in 4.6 mL 0.5 M NaCl (aq.)) for M2.8 before and after the cell cycling tests. (**a**): TMA-TEMPO. (**b**): MV. Scan rate: 200 mV·s-1. Background subtracted before plotting; the fourth scan was used to prepare the comparative graphs.

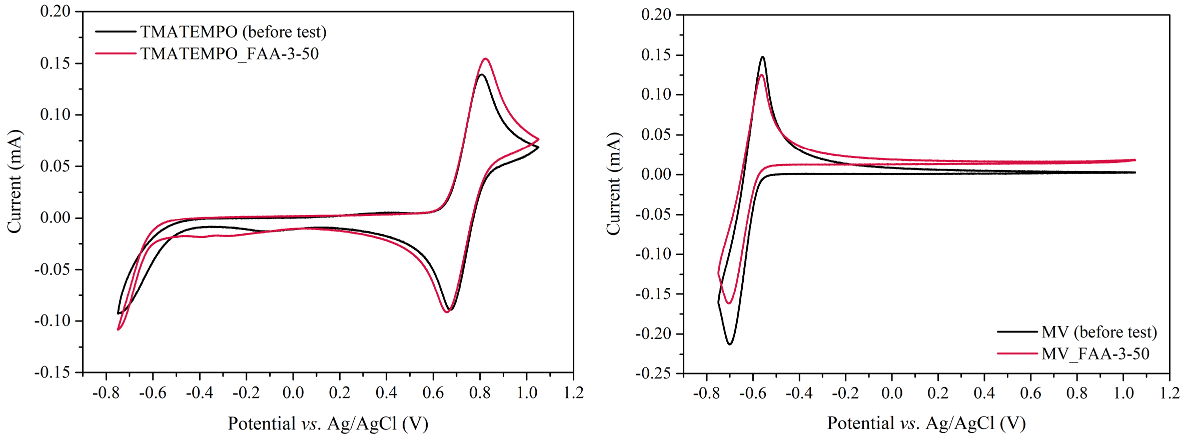

(**a**) (**b**)

Figure S13. Cyclic voltammograms of TMA-TEMPO and MV solutions (100 µL in 4.6 mL 0.5 M NaCl (aq.)) for FAA-3-50® after the cell cycling test. (**a**): TMA-TEMPO. (**b**): MV. Scan rate: 200 mV·s-1. Background subtracted before plotting; the fourth scan was chosen to prepare the comparative graphs.

Micrographs of the AEMs

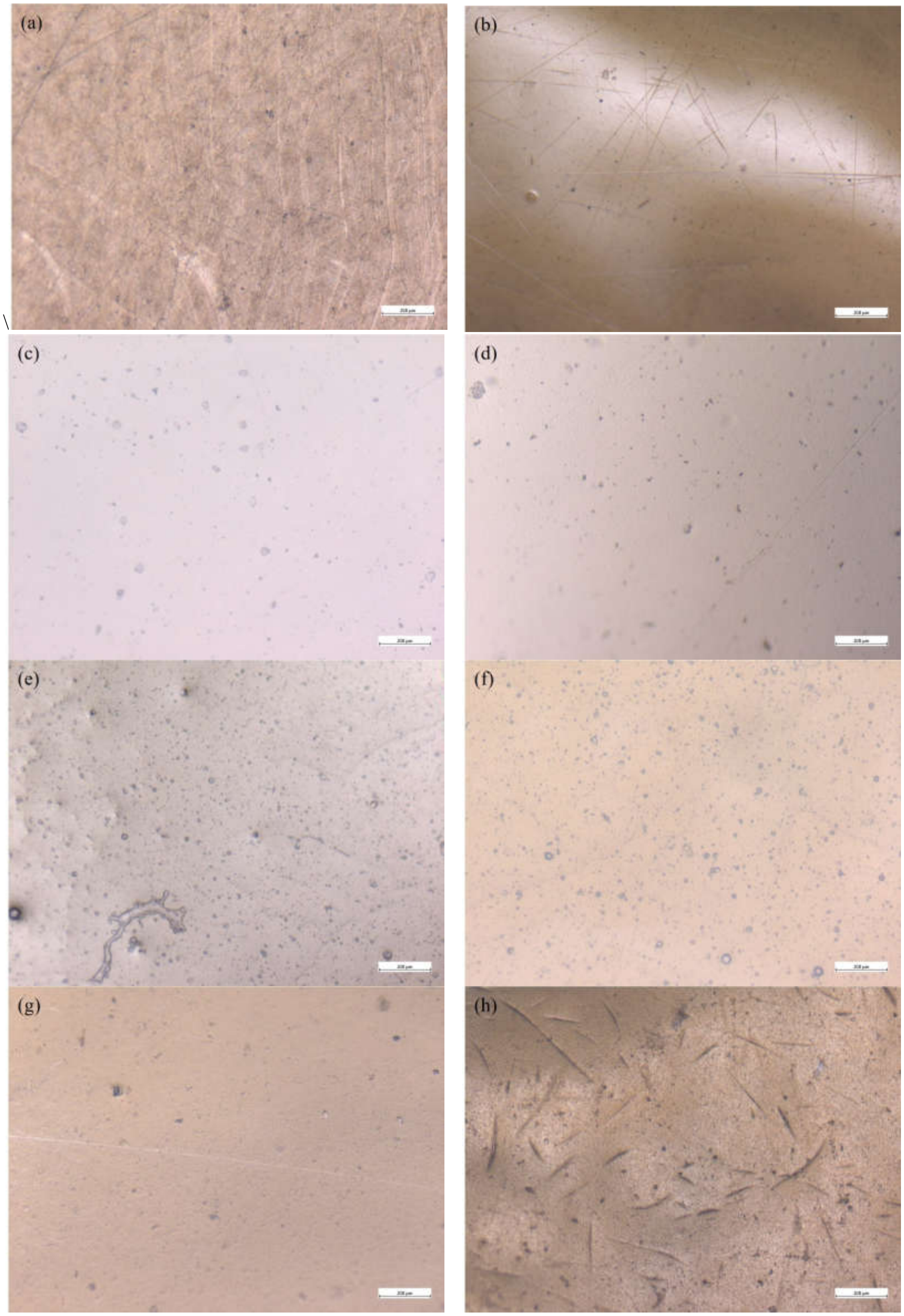

Figure S14. Micrographs of the AEMs before and after cell tests in wet form: (**a**) M1.5-3 before, (**b**) M1.5-3 after, (**c**) M1.7-2 before, (**d**) M1.7-2 after, (**e**) M2.1-2 before, (**f**) M2.1-2 after, (**g**) M2.8-3 before and (**h**) M2.8-3 after. Bar size: 200 μm.
